# FAM3C in circulating tumor-derived extracellular vesicles promotes non-small cell lung cancer growth in secondary sites

**DOI:** 10.7150/thno.72297

**Published:** 2023-01-01

**Authors:** Win Lwin Thuya, Li Ren Kong, Nicholas L. Syn, Ling-Wen Ding, Esther Sok Hwee Cheow, Regina Tong Xin Wong, Tingting Wang, Robby Miguel Wen-Jing Goh, Hongyan Song, Migara K. Jayasinghe, Minh TN Le, Jian Cheng Hu, Wei-Peng Yong, Soo-Chin Lee, Andrea Li-Ann Wong, Gautam Sethi, Huynh The Hung, Paul Chi-Lui Ho, Jean-Paul Thiery, Siu Kwan Sze, Tiannan Guo, Ross A. Soo, Henry Yang, Yaw Chyn Lim, Lingzhi Wang, Boon-Cher Goh

**Affiliations:** 1 Cancer Science Institute of Singapore, National University of Singapore, Singapore 117599.; 2 Department of Pharmacology, Yong Loo Lin School of Medicine, National University of Singapore, Singapore.; 3 Department of Pathology, Yong Loo Lin School of Medicine, National University of Singapore, Singapore.; 4 NUS Centre for Cancer Research (N2CR), Yong Loo Lin School of Medicine, National University of Singapore, Singapore.; 5 Division of Cellular and Molecular Research, National Cancer Centre, Singapore.; 6 Department of Haematology-Oncology, National University Cancer Institute, National University Health System, Singapore.; 7 Department of Pharmacy, Faculty of Science, National University of Singapore, Singapore.; 8 INSERM Unit 1186, Comprehensive Cancer Center, Institut Gustave Roussy, Villejuif, France.; 9 Nanyang Technological University, 60 Nanyang Drive, Singapore 637551, Singapore.; 10 Zhejiang Provincial Laboratory of Life Sciences and Biomedicine, Key Laboratory of Structural Biology of Zhejiang Province, School of Life Sciences, Westlake University, China.; 11 Institute of Basic Medical Sciences, Westlake Institute for Advanced Study, China.; 12 Department of Medicine, Yong Loo Lin School of Medicine, National University of Singapore, Singapore.

**Keywords:** FAM3C, non-small cell lung carcinoma, tumor-derived extracellular vesicles, predictive biomarker, tumor metastasis.

## Abstract

**Rationale**: Metastasis is a complex process with a molecular underpinning that remains unclear. We hypothesize that cargo proteins conducted by extracellular vesicles (EVs) released from tumors may confer growth and metastasis potential on recipient cells. Here, we report that a cytokine-like secreted protein, FAM3C, contributes to late-stage lung tumor progression.

**Methods**: EV protein profiling was conducted with an unbiased proteomic mass spectrometry analysis on non-small cell lung cancer (NSCLC) and normal lung fibroblast cell lines. Expression of FAM3C was confirmed in a panel of NSCLC cell lines, and correlated to the invasive and metastatic potentials. Functional phenotype of endogenous FAM3C and tumor-derived EVs (TDEs) were further investigated using various biological approaches in RNA and protein levels. Metastasis potential of TDEs secreted by FAM3C-overexpressing carcinoma cells was validated in mouse models.

**Results**: Transcriptomic meta-analysis of pan-cancer datasets confirmed the overexpression of FAM3C - a gene encoding for interleukin-like EMT inducer (ILEI) - in NSCLC tumors, with strong association with poor patient prognosis and cancer metastasis. Aberrant expression of FAM3C in lung carcinoma cells enhances cellular transformation and promotes distant lung tumor colonization. In addition, higher FAM3C concentrations were detected in EVs extracted from plasma samples of NSCLC patients compared to those of healthy subjects. More importantly, we defined a hitherto-unknown mode of microenvironmental crosstalk involving FAM3C in EVs, whereby the delivery and uptake of FAM3C via TDEs enhances oncogenic signaling - in recipient cells that phenocopies the cell-endogenous overexpression of FAM3C. The oncogenicity transduced by FAM3C is executed via a novel interaction with the Ras-related protein RalA, triggering the downstream activation of the Src/Stat3 signaling cascade.

**Conclusions**: Our study describes a novel mechanism for FAM3C-driven carcinogenesis and shed light on EV FAM3C as a driver for metastatic lung tumors that could be exploited for cancer therapeutics.

## Introduction

Metastasis of carcinomas represents the end-product of diverse evolutionary processes including the accumulation of oncogenic mutations 1, acquisition of mesenchymal phenotype 2, as well as co-option of diverse cell types within the tumor microenvironment 3. Advanced metastases are often associated with cancer mortality 4 and resistance to therapeutic intervention 5, which then undermine the survival gains of targeted and immune-based therapies particularly in non-small cell lung cancer (NSCLC) 6. NSCLC accounts for the highest cancer mortality worldwide predominantly due to its high metastasis potential. Understanding the molecular mechanisms underpinning these metastatic events and interrupting these pathways is expected to improve clinical outcome among NSCLC patients.

Research on extracellular vesicles (EVs) has gained considerable interest in recent years, and the increased understanding on the pathophysiology of tumor-derived EVs (TDEs) has engendered development of novel diagnostics and therapeutics tools 7. EVs are comprised of exosomes, ectosomes and microvesicles, etc. While they differ in their cellular origin and biogenesis, EVs are constantly secreted into the extracellular microenvironment and could be detected in biofluids such as plasma and urine 7, 8, and may therefore serve as readily available biomarkers for disease diagnosis, prognosis and prediction of treatment effects 9, 10. In addition, disease-associated EVs are intracellular communicators that can be transferred to recipient cells through plasma membrane fusion and endocytosis-mediated internalization. The uptake of these vesicles has been proven to be essential for biological processes that range from neurodevelopment 11 to immune defense mechanism 12, and have direct impact on disease severity.

From the perspective of carcinogenesis, TDEs and their cargo molecules are able to reprogram the neoplastic cells and stroma compartment to facilitate tumor development 13-17. More recently, the discovery that vesicular integrins could determine organ-specific metastasis 18 and facilitate endocytosis of EVs in the recipient cells 19 have placed further emphasis on the biological role of TDEs in reprogramming the tumor microenvironment. This finding challenges the long-standing dogma that autocrine and paracrine signaling are regulated mainly through secreted proteins. We and others have previously articulated a conceptual framework for the mechanisms of EVs-mediated metastasis 7, 20, 21, which involves (i) paracrine and autocrine signaling for the intracellular induction of epithelial-mesenchymal transition (EMT), (ii) distant cell-to-cell communication that facilitate metastatic colonization, and (iii) alteration of the tumor microenvironment to enable escape from anti-tumor immuno-surveillance. While the multifarious roles of TDEs are established in cell-cell communication and cancer progression/metastases, the specific molecules mediating these effects need further elucidation if we are to find therapeutically tractable solutions.

In the present study, by coupling mass-spectrometry-based proteomics and biochemical assays in lung cancer cells validated with patient's plasma samples, we identify FAM3C, a secreted interleukin-like EMT inducer (ILEI), is an oncogenic cargo protein encapsulated within TDEs through which cell-cell growth signals are potentially communicated. FAM3C is a potent inducer of tumor metastasis which regulates EMT through TGF-β signaling 22-24, and is associated with poorer prognosis in several cancer types 17, 25, but its role in NSCLC remains unclear. Functionally, we show that intracellular expression of FAM3C in NSCLC cells not only promotes cancer cell growth and invasion *in vitro* and *in vivo*, but extracellular transmission of FAM3C-containing TDEs can also induce aggressive phenotypes in recipient cells resembling those of cell autonomous FAM3C overexpression. Mechanistically, we establish that TDE derived FAM3C interacts with Ras-related protein RalA to trigger oncogenic signals.

## Materials and methods

### Cell Culture

Lung carcinoma cell lines utilized in this study were purchased from the American Type Culture Collection (ATCC) (Manassas, VA, USA); they include HCC827, H1975, H1650, H226, A549, H1299, SKMES-1, Calu-1, H2172, ChaGo-K, H520, H2170 and growth conditions were detailed in Supplementary Information.

### EVs isolation

Cells were cultured in T75 cell culture flasks until 80-90% confluency. The culture media were discarded and the cells were washed with 1x phosphate- buffered saline (PBS) at least 3 times to remove all bovine EVs. Then, the cells were cultured in serum-free RPMI-1640 or MEM supplemented with 1% L-glutamine and 1% penicillin/streptomycin. EVs isolation conditions were detailed in Supplementary Information.

### Scanning Electron Microscopy (SEM)

EVs samples were first fixed in 2.5% Glutaraldehyde (Electron Microscopy Services, Hatfield, PA, USA) in 0.1M Sodium Cacodylate buffer, pH 7.4 (Electron Microscopy Services, Hatfield, PA, USA) for 60 min, and rinsed in 0.1M Sodium Cacodylate buffer (3 x 10 min) at 25°C. The process was detailed in Supplementary Information.

### Characterization of extracellular vesicles by DLS

The size distribution of EVs isolated from plasma samples were determined using Zetasizer Nano ZS90 (Malvern Instruments Ltd, Malvern, UK) and the light scattering was measured. Each sample was measured at dispersant refractive index of 1.330 and viscosity of 0.8872 cP at 25°C, and the size distribution of each sample was recorded through a sequence of 12×10 s recordings which were repeated three times.

### Western blot analysis

Cells were lysed in PIERCE™ RIPA lysis buffer, supplemented with Halt™ protease and phosphatase inhibitor cocktail (Thermo Fisher Scientific). The proteins were separated on 8-12% SDS-PAGE gels and the electrophoresed gels were transferred into PVDF membranes. Western blotting conditions were detailed in Supplementary Information.

### Proteomics sample processing and analysis

Briefly, extracted EV proteins were separated in gel digestion, following tryptic digestion into peptides which were subsequently analyzed by Linear Trap Quadrupole Fourier Transform (LTQ-FT) mass spectrometry. The sample processing and analysis were detailed in Supplementary Information.

### RT-qPCR analysis

Reverse transcription-quantitative polymerase chain reaction was performed to quantify and validate RNA sequencing results, using GAPDH as a loading control. For primers used, see Table S3.

### Immunohistochemistry (IHC) staining and scoring

The NSCLC tissue microarray comprising 45 cases of NSCLC tissues, 47 cases of tumor adjacent normal tissues and 8 cases of normal lung tissues was obtained from US Biomax, Inc. IHC conditions and description of the scoring criteria for FAM3C expression were detailed in Supplementary Information.

### FAM3C plasmid constructs and lentiviruses transduction

Human FAM3C MISSION shRNA Lentiviral Transduction Particles and MISSION TRC2 Control Vector pLKO-puro TurboGFP shRNA were obtained from Sigma. The sequence for FAM3C shRNA was CCGGCTTGGTGTGTGCATGAGTATTCTCGAGAATACTCATGCACACACCAAGTTTTTG. Lentiviruses transduction procedure was detailed in Supplementary Information.

### Recombinant human FAM3C protein treatment

Recombinant human FAM3C protein was purchased from MyBiosource (Catalog #: MBS204141). NSCLC cells including H2170, A549 and SKMES-1 were treated with 10 ng/mL FAM3C recombinant protein for 48 hours before experimental analyses.

### *In vitro* invasion assay

Cell invasion capability was performed using a Corning® BioCoat™ Matrigel Invasion Chambers (#354480). Cell invasion procedure was detailed in Supplementary Information.

### *In vitro* cell migration assay

The cells were cultured until they reached 90-95% confluence. A scratch was created through the monolayer in each well using a 300ul pipette tip. Monolayer was washed with 1 x PBS and media was changed daily. Cell movement into the wound area was monitored and photographed at 0 hour and 24 hours using Zeiss Axio Vert A1 inverted microscope (Carl Zeiss Microscopy GmbH, Oberkochen, Germany).

### Anchorage-dependent and -independent colony formation assays

For anchorage-dependent assay, human lung carcinoma (SKMES-1, A549 and H2170) control, FAM3C-overexpressed and shFAM3C-knockdown cells were seeded at a density of 5,000 cells/well in 6 well plates. The anchorage-dependent and independent colony assays were detailed in Supplementary Information.

### Extracellular vesicles uptake by NSCLC cells

The fluorescent reagent Exo-Green (System Biosciences) was utilized to label the protein component of H2170 lung carcinoma cell-derived EVs. The process was detailed in Supplementary Information.

### Duolink Proximity Ligation in situ Assay (P-LISA)

For Duolink in situ, all incubations were performed in a 37 °C humidity chamber. Cells were plated onto glass coverslips and fixed with 4% paraformaldehyde at room temperature for 30 min. PLA staining was conducted according to the manufacturer's protocol (Duolink, #DUO92101). Preparations were mounted with prolong gold antifade reagent with DAPI (Life Technologies). PLA images were analyzed using ImageJ software (NIH), expressed with the mean PLA/cell intensity ± SEM.

### Phosphokinase antibody array

Human phosphokinase antibody array kits were obtained from R&D Systems® to analyze the phosphorylation profiles of kinases according to manufacturer's instructions (https://www.rndsystems.com/resources/technical/assays-analytes-represented-human-phospho-kinase-array-kit).

### RNA sequencing analysis

Total RNA was isolated using Qiagen RNeasy Kit and sequenced using Illumina Hiseq4000. Differently expressed genes [with a threshold log2 (fold change) ≥ 0.5] were analyzed using Gene Set enrichment analysis (GSEA) and ConsensusPathDB for pathway enrichment analysis.

### TCGA data of NSCLC

The TCGA pan cancer RNA sequencing data were analyzed using Gene Expression Profiling Interactive Analysis (GEPIA). The expression level of FAM3C in different sets of lung cancer patient cohorts was retrieved and analyzed using GEO gene expression database.

### *In vivo* lung tumor colonization assay

The animal experiments were approved by ethics board from the SingHealth Institutional Animal Care and Use Committee (IACUC) at the National Cancer Centre Singapore and Singapore General Hospital. All mice were maintained according to the "Guide for the Care and Use of Laboratory Animals" published by National Institute of Health, USA. They were provided with sterilized food and water ad libitum, and housed in negative pressure isolators with 12h light/dark cycles.

Metastatic ability of FAM3C was determined by tail vein injection of Control-Vector cells, FAM3C knockdown and over-expressed A549 cells into mice. 1×10^6^ cells/100 µl PBS were injected into tail vein of the respective mice. After 2 months, the mice were euthanized; and their lungs were harvested, fixed in buffered formalin and stained with Bouin's solution. After that, the lungs were destained prior to processing into paraffin blocks and 4µm thick sections prepared for IHC staining.

For determination of metastasis potential of tumor-derived EVs in mice, 10 μg of control A549 and FAM3C-overexpressing A549 cells-derived EVs were injected intravenously via the tail vein into mice twice a week for two weeks prior to primary cells administration. At the beginning of week 3, the mice were inoculated with A549 primary cells together with tumor-derived EVs via tail vein injection. EVs were injected subsequently twice a week for an additional 5 weeks. Mice were sacrificed and lung organs were harvested to investigate the efficiency of tumor-derived EVs to induce tumour cell engraftment. Lungs were fixed in formalin, processed into paraffin blocks and tissue sections were stained for pan-cytokeratin and FAM3C expression as described above.

## Statistical analysis

All data analysis was conducted using Graph Pad Prism 9 software (GraphPad Software, La Jolla, CA, USA). All results are reported as mean ± SEM. Statistical differences between two groups were evaluated using two-sided paired Student's t-test, while comparison between multiple groups was conducted using one-way ANOVA. Differences with a *p* value < 0.05 were considered statistically significant. The non-parametric one-tailed Mann-Whitney U test was carried out for the mouse lung tumor nodule analyses.

## Results

### FAM3C is expressed in TDEs released from NSCLC tumors and its expression correlates with poor prognosis

Our central hypothesis is that EV proteins may drive oncogenic signaling within the tumor microenvironment. Proteomic quantification was performed on EVs derived from a panel of NSCLC cell lines (HCC827, H1650, and H520) and fibroblast cell line using label-free LTQ-FT mass spectrometry. EVs were purified using differential centrifugation followed by ultracentrifugation - an established method for EVs isolation (**Figure S1A**). To verify the characteristics of isolated EVs, we showed - by dynamic light scattering analysis - that isolated EVs formed a bell-shaped size distribution profile with average size of 130.4 nm (**Figure S1B**). The purity of the extracted EVs were validated with the universal EVs markers (TSG101, CD63, and CD9), without the contamination of the cis-Golgi matrix protein (GM130) and marker for endoplasmic reticulum (Calnexin) (**Figure S1C**).

**Figure 1:**
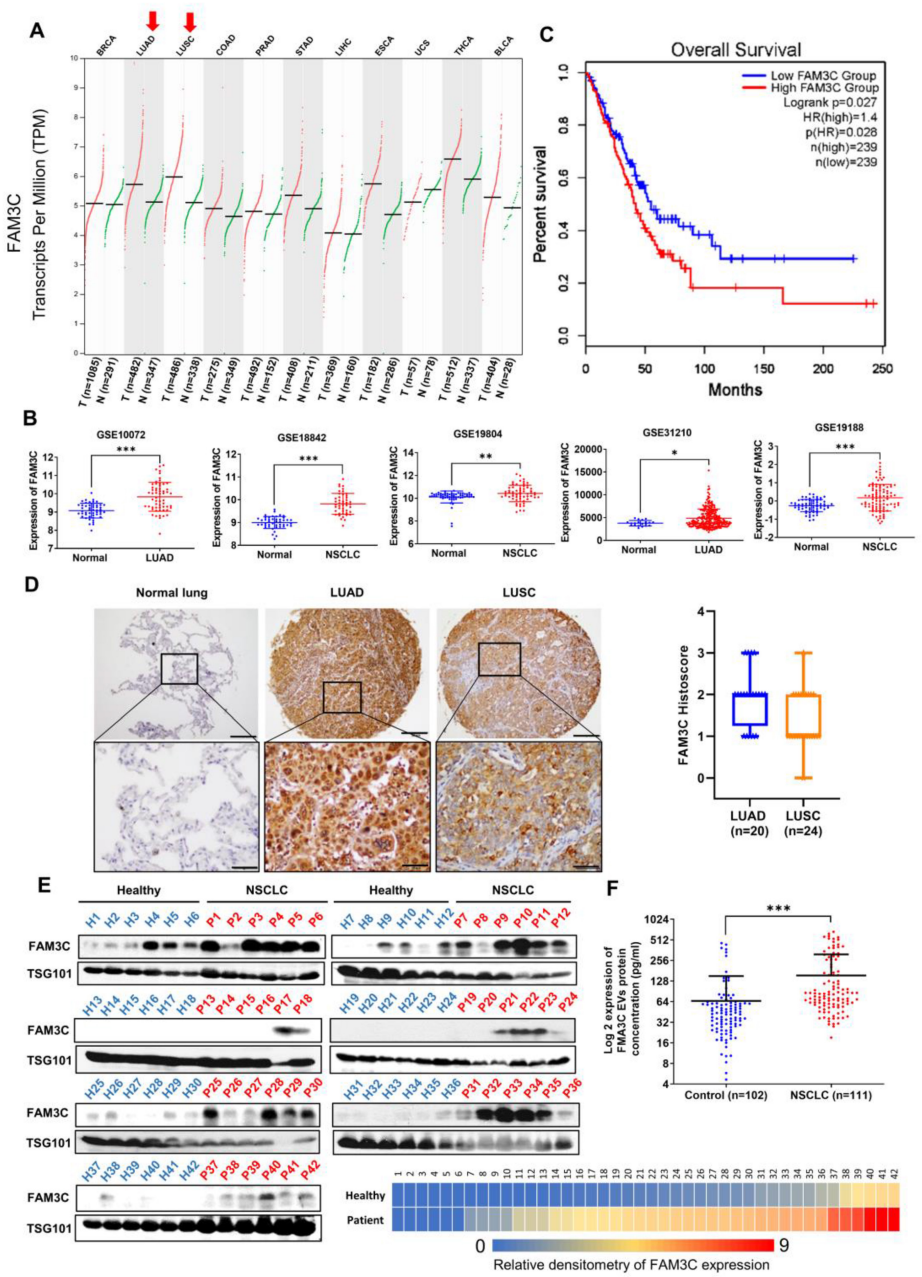
** FAM3C expression is associated with poorer prognosis in lung carcinomas. (A)** Analysis of *FAM3C* mRNA expression in 11 different types of human cancer (TCGA PanCancer Atlas Studies from cBioPortal database). Red, tumor samples; green, normal tissue. BRCA, breast carcinoma; LUAD, lung adenocarcinoma, LUSC, lung squamous cell carcinoma; COAD, colon adenocarcinoma; PRAD, prostate adenocarcinoma; STAD, stomach adenocarcinoma; LIHC, liver hepatocellular carcinoma; ESCA, esophageal carcinoma; UCS, uterine carcinosarcoma; THCA, thyroid carcinoma; BLCA, bladder carcinoma. **(B)** *FAM3C* mRNA expressions in five independent lung carcinoma datasets: GSE10072, GSE18842, GSE19804, GSE31210 and GSE19188. LUAD, lung adenocarcinoma; NSCLC, non-small cell lung carcinoma. **(C)** Kaplan-Meier survival plot showing prognosis of patients stratified by *FAM3C* expression (Data retrieved from gene expression profiling interactive analysis). **(D)** NSCLC tissue microarray with 45 cases of NSCLC and 47 cases of tumor adjacent normal tissue including 8 cases of normal lung tissues (US Biomax). The representative images of FAM3C expression in normal lung tissues, LUAD and LUSC (left panel). Scale bar, 100 μM. Box plot showing the histoscore for FAM3C expression in TMA for adenocarcinoma and squamous cell carcinomas (right panel). **(E)** Quantitative analysis by Western blot for FAM3C expression in plasma EVs in NSCLC patients (n=42) and healthy controls (n=42). The heatmap in the bottom right panel represents relative density of the Western blot bands of FAM3C normalized to the densitometry values of internal exosomal marker, TSG101 compared with control (*p* value <0.0001). **(F)** Scatter plot of EV FAM3C concentrations in plasma samples based on the ELISA results of the NSCLC group (n=102) and healthy control group (n=111).

From the purified EVs (**Figure S1C**), we identified a total of 2,658 distinct proteins in EVs shed by NSCLC cells (HCC827, H1650 and H520) and IMR90 cells at a false discovery rate (FDR) of 1%, with 1,369 proteins found to be specific to NSCLC (**Figure S1D, left**). Among the EV proteins detected in the three NSCLC cell lines (2,371), 709 proteins were commonly shared by all cancer cell lines (**Figure S1D, right**). Among the top 20 differentially expressed proteins with a fold-change of > 6 (**Figure S1E, Table S1**), we identified FAM3C, a small secreted protein with strong EMT inducing property. To investigate the clinical relevance of this finding, we successfully purified and validated EVs from plasma samples of NSCLC patients and healthy individuals (**Figure S2A**), and measured EV FAM3C expression by immunoblotting in 84 plasma samples (**Figure 1E**) and ELISA in 213 plasma samples (**Figure 1F**), demonstrating significantly higher FAM3C in cancer patients. More importantly, higher FAM3C concentrations in the EVs extracted from patient plasma was associated with more advanced stages of NSCLC (**Figure S2B**). In addition, the other top hits identified from the cell line-derived EVs (ANXA4, RPS9, RPLP1, and PSME3) were detected in plasma EVs as well (**Figure S2C-F**), thus validating the robustness of our proteomic approach. Given the known role of FAM3C in promoting EMT in a few cancers, we hypothesized that FAM3C carried on TDEs could promote proliferation of NSCLC at secondary sites.

To unravel the clinical significance of FAM3C in carcinomas, we began by delineating its clinicopathological and molecular correlation by leveraging on publicly available transcriptomic datasets as well as immunohistochemical evaluation of FAM3C expression in tissue microarrays. Notably, *FAM3C* expression is elevated in malignant lung tissues (lung adenocarcinoma, LUAD; and lung squamous cell carcinoma, LUSC) compared to the non-malignant tissues based on The Cancer Genome Atlas (TCGA) database (**Figure 1A**). Furthermore, significantly higher mRNA expression of *FAM3C* in carcinoma tissues was observed as compared to the normal lung counterparts based on five independent lung carcinoma datasets (**Figure 1B**). Using one-stage meta-analysis on patient survival data from these datasets, we found high *FAM3C* mRNA expression (stratified at 239 for both high and low median) to be correlated with poorer prognosis (marginal HR = 1.40; 95% CI: 1.04-1.89; *p* = 0.028) (**Figure 1C**), thereby suggesting its pertinence in NSCLC.

To confirm the specificity of FAM3C in tumor cells, its expression profile across normal lung tissues and early to late stage carcinomas was assessed in a scRNA-Seq readout of 44 LUAD patients 26. Of note, *FAM3C* is scarcely detected in non-transformed epithelial tissues and lymph nodes (**Figure S3A-B**). In contrast, *FAM3C* is significantly elevated in the epithelial compartment of the cancerous tissues (**Figure S3C**), with an enrichment of FAM3C-positive populations in the lymph node and brain metastases (**Figure S3D-E**). By focusing on the epithelial compartments of the primary tumors and metastases, there was an increase of FAM3C expression in most of the tumor clusters of the metastases (**Figure S3F-G**). Collectively, these analyses suggested that FAM3C is likely of tumor origin and may play a significant role in the disease progression of lung carcinoma metastasis.

Semi-quantitative measurement of FAM3C using immunohistochemical staining was performed on 45 cases of NSCLC, 47 cases of tumor-adjacent normal tissue, and 8 cases of normal lung tissue (**Table S2**). Microscopic analyses of the tissue histology revealed that FAM3C is predominantly localized in the cytosol, with strong expression detected in the cancer cells (**Figure 1D**). Interestingly, all normal lung parenchyma showed negative staining (n=0/10, 0.0%), while 17 of 47 of tumor-adjacent “normal” tissue (36.2%), and 44 of 45 of NSCLC samples (97.2%) were stained positive (Pearson's χ^2^ = 50.28, *p*=1.2
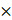
10^-11^). FAM3C expression was detected in both LUAD and LUSC (**Figure 1D, left**), although more variable staining intensities were seen within the LUSC subtype (**Figure 1D, right**). Collectively, both transcriptomic and immunohistochemical data confirmed the specificity of FAM3C in lung tumors and it is associated with poorer prognosis.

### Aberrant FAM3C promotes NSCLC cell motility and anchorage-independent growth

**Figure 2:**
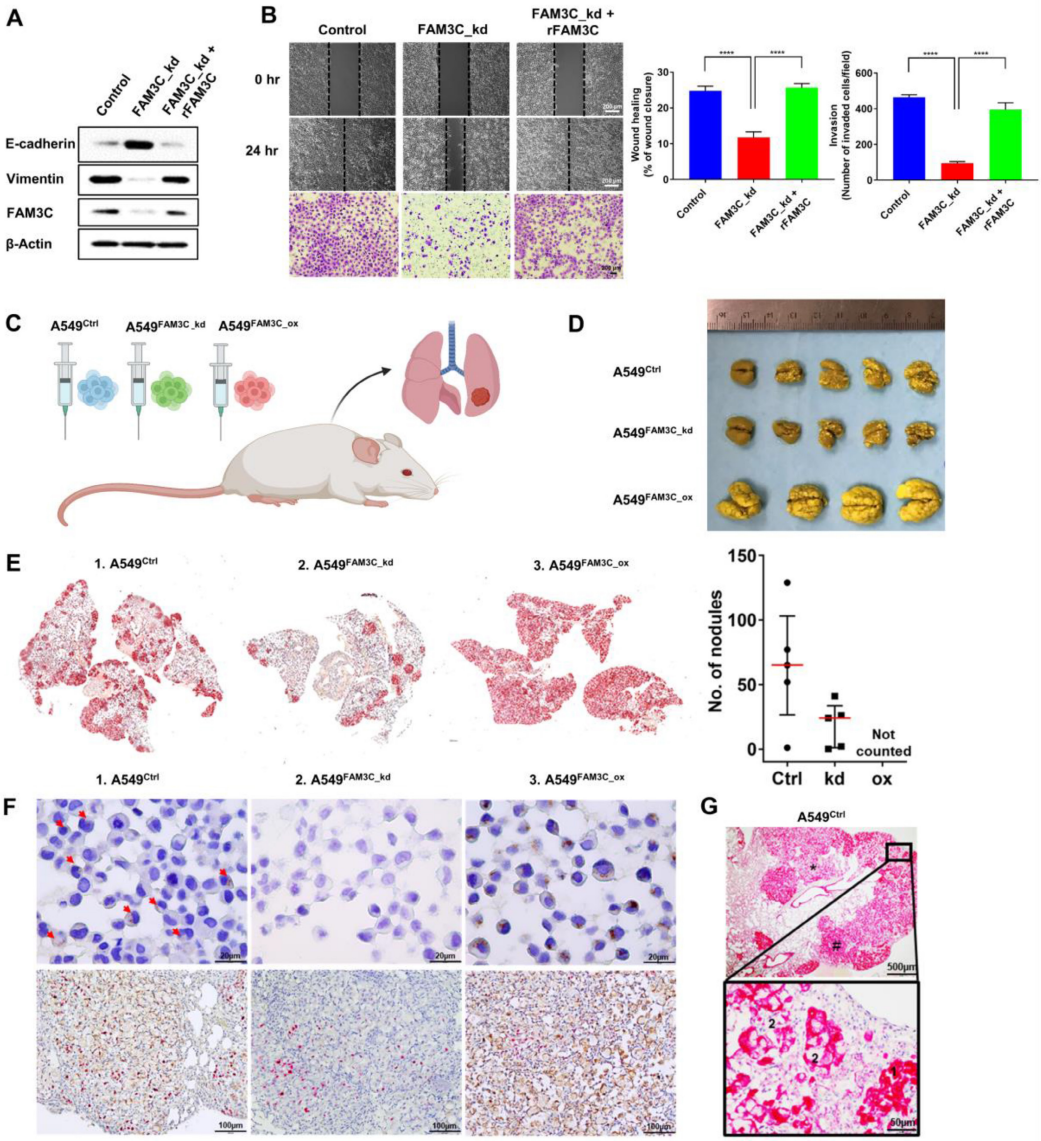
** FAM3C impacts invasiveness of lung carcinoma cells. (A)** A549 FAM3C knockdown cells (FAM3C_kd) were treated with human recombinant FAM3C (rFAM3C) protein and expression of FAM3C and EMT markers (E-cadherin and Vimentin) were analyzed by Western blot analysis. **(B)** The effect of recombinant FAM3C on cell motility was examined by Matrigel invasion and wound healing assays. The data presented were from three independent experiments, each with three replicates and are represented as mean ± SEM. *** *p* < 0.001. **(C-E)** FAM3C promotes lung colonization *in vivo*. **(C)** A549 parental cells (Ctrl), A549-FAM3C knockdown (FAM3C_kd) cells, and A549-FAM3C overexpressed (FAM3C_ox) cells were injected intravenously into five nude mice per cell type. Mice were sacrificed and analyzed for the presence of lung metastasis. **(D)** Macroscopic images of Bouin's stained lungs from five individual mice from each group. Visible lung tumor colonies appear as yellowish white spots on the lung surface. **(E)** Representative images of whole lung sections stained with cytokeratin (CK). Bright red clusters are A549 lung colonization. Counts for CK-positive nodules were tabulated as median ± interquartile range (n = 5). **(F)** (upper panels) Representative images of FAM3C expression (brown) in the A549 Ctrl cells, FAM3C_kd and FAM3C_ox transduced cells, red arrows indicate FAM3C-positive A549 Ctrl cells; (bottom panels) FAM3C (brown)-Ki-67 (red) double staining of lung sections of A549 Ctrl, FAM3C_kd and FAM3C_ox tumors. **(G)** Representative images of A549 Ctrl lung tumor section stained with cytokeratin (red). Box, 40X magnification of indicated area.

Endogenous FAM3C expression has been reported to drive metastatic outgrowth via EMT induction in breast and gastric carcinomas 22, 24, 27. However, its pathophysiologic role in NSCLC has not been investigated in depth. Here, we screened FAM3C protein expression in 12 NSCLC cell lines. FAM3C expression was low in the normal lung fibroblast (IMR90) cell line. In contrast, most of adenocarcinoma or squamous cell lines showed high FAM3C content (**Figure S4A),** which is consistent with the scRNA-Seq database** (Figure S3A).** To delineate the role of FAM3C in NSCLC, we modulated its expression in the FAM3C-high SKMES-1 (using shRNA-mediated knockdown), FAM3C-low H2170 (using plasmid-based overexpression), and FAM3C-intermediate A549 cell lines (knockdown or overexpression). In agreement with previous reports from others, our data demonstrated that FAM3C is a strong inducer of cell motility and malignancy. FAM3C-knockdown (FAM3C_kd) in SKMES-1 effectively increased E-cadherin expression and reduced vimentin (**Figure S4B**) - both quintessential EMT-related markers and reduced cell migration (**Figure S4C**), invasion (**Figure S4D**), and clonogenicity (**Figure S4E**). Reciprocally, FAM3C-overexpression (FAM3C_ox) in H2170 suppressed E-cadherin while induced vimentin, and concomitantly induced invasiveness, migration and anchorage-independent growth (**Figure S4B-E, middle**). In consonance with experiments with SKMES-1 and H2170 cells, knockdown and stable overexpression of FAM3C in A549 was accompanied by downregulation and upregulation of E-cadherin and vimentin respectively (**Figure S4B, right**). Modulating FAM3C levels in A549 cells had significant effect in cell motility and were mirrored by statistically significant changes in anchorage-dependent and anchorage-independent tumorigenesis both in terms of colony counts and size of colonies (**Figure S4C-E, right**). Furthermore, FAM3C oncogenic activity was confirmed via rescue experiments in FAM3C_kd A549 cells by co-incubation with recombinant FAM3C protein (**Figure 2A-B**). Taken together, the afore set of observations convincingly established the role of FAM3C in imparting NSCLC cells with pro-invasive and tumorigenic properties.

### FAM3C promotes lung carcinoma cell colonization *in vivo*

Having shown that FAM3C promotes EMT and cancer aggressiveness *in vitro*, we wonder if changes in intracellular FAM3C translates to any appreciable effects *in vivo*. We established mouse models of lung colonization through tail vein injection of parental A549 (Ctrl) cells or the modified counterparts with FAM3C knockdown (FAM3C_kd) and overexpression (FAM3C_ox) (**Figure 2C**). Resected lungs were washed and stained in Bouin's solution, and both microscopy and macroscopy analyses revealed gross disparities in intrapulmonary tumor burden of mice that were positively correlated to FAM3C status. Tumor nodules appeared as yellowish white spots on the lungs of mice injected with A549 parental and FAM3C_kd cells (**Figure 2D**). In contrast, A549 FAM3C_ox cells have colonized the whole lung, as demonstrated by the enlarged lung size and the yellowish white coloration of the entire lung (**Figure 2D**). Pan-cytokeratin staining (red) revealed the presence of tumor nodules in the lungs of mice injected with A549 parental and FAM3C_kd cells (**Figure 2E**); however, the number of nodules in the A549 FAM3C_kd group was significantly fewer than that of the parental cell group (**Figure 2E, right panel**). The nodules in the lungs of mice injected with A549 FAM3C_ox cells cannot be counted because they were extremely aggressive and had colonized and defaced the entire lung parenchyma of the host animals.

We next validated FAM3C expression in the parental and transduced cell lines; and their respective lung tumors (**Figure 2F upper panels**). While only a small proportion of the A549 Ctrl cells show weak FAM3C expression (red arrows), strong FAM3C expression was detected in ~70% of the A549 FAM3C_ox cells. As expected, the cytokeratin positive FAM3C_kd derived tumor nodules exhibit negligible FAM3C expression (**Figure S5A; Figure 2F middle panels**). In contrast, the FAM3C expression in the FAM3C_ox derived lung tumor nodules were more intense than that of A549 parental cell origin (**Figure 2F**).

While some tumor nodules or part of some nodules in the lung sections appear to be tightly packed and intensely stained (**Figure 2G, #**); the rest exhibit an open and lacey appearance with less intense cytokeratin staining (Figure 2G, *). Further examination at high magnification show that darkly stained nodules are made up small cancer cells with strong cytoplasmic and membrane cytokeratin staining (**1, Figure 2G** and **Figure S5A**), while the cells in lighter nodules often appeared thinly spread and elongated (**2, Figure 2G and Figure S5A**). More intriguingly, positive nuclear Ki67 staining (red), indicating the presence of proliferating cells, is consistently found within the clusters of small cells (**Figure 2F, 1 Figure 5B**) but rarely amongst the larger spread cells (**Figure 2F, 3 Figure S5B**). FAM3C expression in the A549 parental and FAM3C_ox tumor nodules appear to be unipolar or perinulcear in these small tightly packed carcinoma cells (**Figure 2F upper panels; 1, Figure S5B, red arrows**) and as punctate cytoplasmic positivity proximal to the nuclei in the larger spread carcinoma cells (**2**, **Figure S5B, black arrows**).

Taken together these observations suggest that the small tightly pack cells are the newly divided and actively proliferating cells; and the thinly spread and elongated cells are cells that appear to have lost the ability to divide. At this juncture, we are unclear of the reason for this loss. As this observed change in cell morphology and proliferation potential are common features in the A549 parental/Ctrl (**Figure 2G**), FAM3C_kd (**Figure S5A**) and FAM3C_ox (not shown) lung sections, we can conclude that this is an inherent characteristic of the A549 lung cancer cell line independent of the cells' FAM3C expression status. In contrast, the aggressiveness of the FAM3C_ox cancer cells in near complete colonization the host lungs compared to the A549 ctrl and FAM3C_kd cells further supports our hypothesis that FAM3C is a potent inducer of cancer metastasis and disease progression.

### TDEs mediate cell-to-cell transfer of FAM3C protein and increase migration and invasion

**Figure 3:**
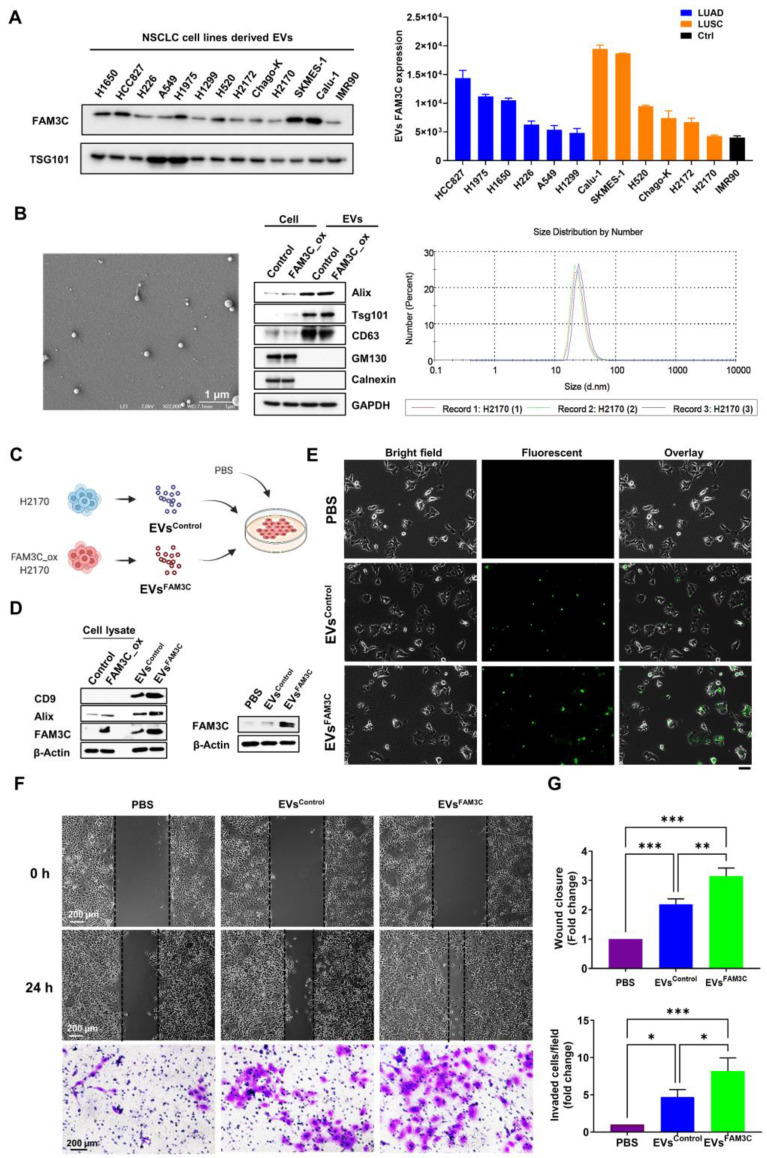
** Tumor-derived EVs containing FAM3C promote invasiveness of lung carcinoma cells *in vitro*. (A)** Western blot analysis of FAM3C expression in cell lines-derived EVs isolated from 12 NSCLC cell lines compared to normal lung fibroblast (NLF) cell line (IMR90). The histogram represents expression levels of FAM3C in EVs derived from normal lung fibroblast (black), LUAD (blue) and LUSC (orange) after normalization against TSG101. **(B)** Morphological characterization of EVs were performed by scanning electron microscopy at 60k magnification (SEM). Scale bar, 1 μM (left panel). Western blot analysis of three positive (+) EVs markers (Alix, Tsg101 and CD63), and two EV negative (-) markers (GM130 and Calnexin) in cell lysates and EVs isolated from the culture media of the respective cell lines (middle panel). GAPDH served as loading control. Size distribution of H2170 cell-derived EVs was assessed by the Zetasizer Nano ZS90 (right panel). **(C)** Experimental set up for EVs uptake by lung carcinoma cells. The EVs isolated from H2170 control (EVs^Control^) and FAM3C-overexpressed (EVs^FAM3C^) cells were added into culture medium of parental H2170 cells to evaluate for metastasis potential. **(D)** Western blot analysis of EV markers (CD9 and Alix) and FAM3C expression in cell lysates and EVs isolated from the culture media of the H2170 control and overexpressed FAM3C cells (left panel). Western blot analysis of FAM3C expression in H2170 cells treated with PBS, 25 μg/mL of EVs^Control^ and 25 μg/mL of EVs^FAM3C^ (right panel). **(E)** Bright field and fluorescent images of cells 6 hours post EVs administration. H2170 cells were treated either with PBS, 25 μg/mL of EVs^Control^ or 25 μg/mL of EVs^FAM3C^, and labelled with Exo-Green. Representative images were shown. Scale bar: 100 μM. **(F-G)** Effects of PBS, EVs^Control^ or EVs^FAM3C^ are assessed with wound healing (top) and Matrigel invasion assays (bottom) in H2170 cells after treatment with 25 μg/mL of respective EVs. **(F)** Representative images are shown. **(G)** Data are presented as mean of three independent experiments ± SEM. * *p* < 0.05, ** *p* < 0.01 and *** *p* < 0.001.

In recent years, compelling evidences have indicated that TDEs may serve as paracrine and autocrine messengers within the tumor microenvironment to induce tumor drug resistance 15, metastasis 13, 14, 16, 17, and malignant growth 28. While FAM3C was first characterized as a secreted protein, we showed here that FAM3C was also enriched in TDEs released by the panel of NSCLC cell lines (**Figure 3A**), with strong concordance to the endogenous protein expression (**Figure S4A**). Accordingly, we postulate here that FAM3C-containing EVs may promote intercellular crosstalk within the tumor microenvironment to drive tumor malignancy and metastases. By Here, we proposed to investigate whether (i) the secreted EVs can be internalized by neighboring neoplastic cells, (ii) if their uptake is sufficient to confer invasive cue in recipient cells, and (iii) whether TDEs secreted by carcinoma cells could hasten disease progression *in vivo*. To probe the first two questions, EVs shed by FAM3C-overexpressed H2170 and A549 cells (EVs^FAM3C^) and their respective control cells (EVs^Control^) were isolated. The particle size of the isolated EVs were in the range of 30-100 nm (**Figure 3B, left and right**), and the purity was confirmed by the strong EV surface markers (Alix, Tsg101 and CD63) relative to cell lysates (**Figure 3B, middle**) (**Figure S6B**). On the contrary, two EV negative (-) markers (GM130 and Calnexin) were highly expressed in cell lysates but undetectable in EVs. We showed that EV FAM3C protein was protected by proteinase k treatment; whereas disruption of the surface membrane of EV led to degradation of FAM3C, suggesting that EV encapsulation could enhance the stability of the cargo molecules (**Figure S6C**). Next, the EVs isolated from H2170 and A549 control (EVs^Control^) and FAM3C-overexpressed (EVs^FAM3C^) cells were incubated in their parental cells (**Figure 3C**) (**Figure S6E**), and the cellular uptake was confirmed by probing for intracellular FAM3C expression (**Figure 3D**). Moreover, direct imaging of H2170 cells cultured with fluorescent-labelled EVs^FAM3C^ showed punctate fluorescence signals (**Figure 3E**), proving that successful uptake of EVs may facilitate transfer of cargo molecules. Next, we asked whether the internalization of EVs^FAM3C^ elicits metastatic properties in recipient cells as compared to EVs^Control^ treatment. We observed a more efficient wound closure and invasion in H2170 and A549 cells incubated with EVs^FAM3C^ as compared to EVs^Control^ (**Figure 3F-G**) (**Figure S6F-G**).

As a secreted protein, FAM3C is reported to bind to the leukemia inhibitory factor receptor (LIFR) to drive oncogenic signaling 29. As such, we further investigated the effect of LIFR on FAM3C intracellular uptake. The results of our study indicated that there was no association of LIFR with FAM3C cellular expression in NSCLC (**Figure S9A-B**). While rFAM3C and EVs enhanced pro-migratory and pro-invasive cue of both in control and LIFR knockdown cells (**Figure S9C-E**), the EVs exhibited greater effect than rFAM3C. Taken together, our data strongly indicates that TDEs secreted by FAM3C-overexpressing NSCLC cells can indeed evoke biochemical and phenotypic changes in recipient cells.

### FAM3C containing TDEs promote distant colony formation

**Figure 4:**
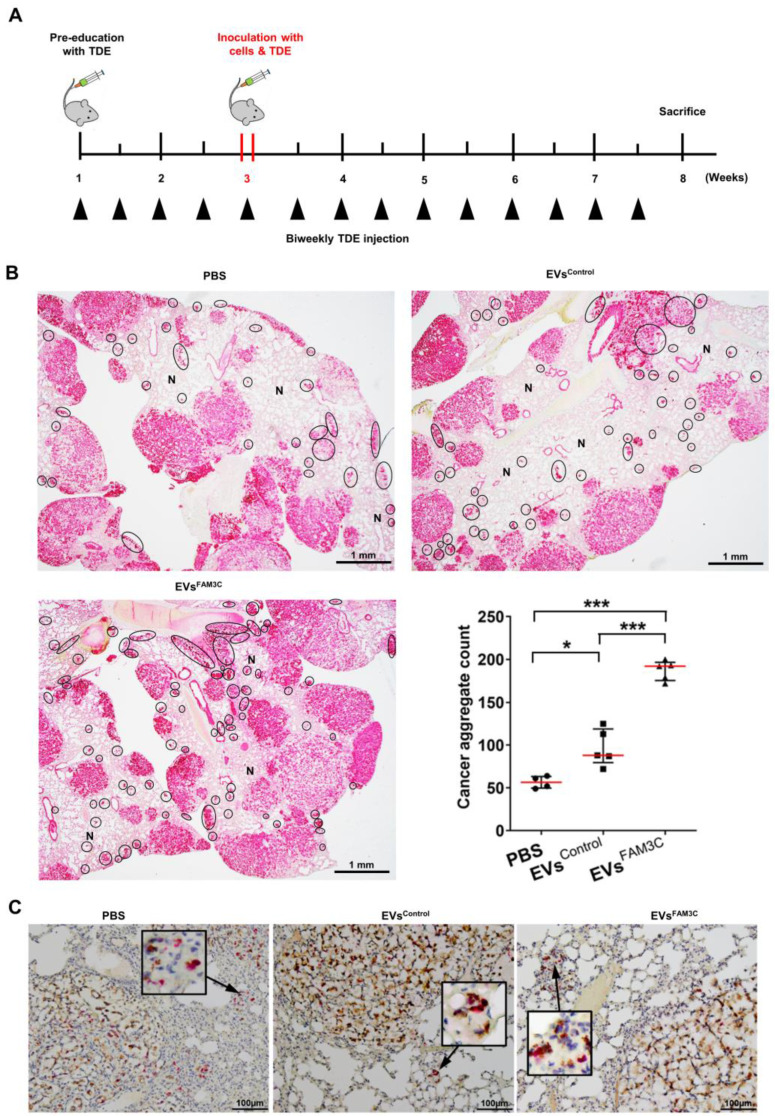
** Tumor-derived EVs containing FAM3C trigger aggressiveness of lung carcinoma cells *in vivo*. (A)** Tail vein injection of A549 lung carcinoma cells in mouse model after 2 weeks pre-educated with PBS, EVs^Control^ or EVs^FAM3C^. Animals were sacrificed after week 7. **(B)** Representative images of cytokeratin (red) stained tissues. Tumor nodules are stained pink with lacy appearance. Black circle, small tumor cell aggregates highlighted as dark red clusters; N=normal lung parenchyma. Counts for tumor aggregates were tabulated as median ± interquartile range (n=4 for PBS, n=5 for EVs^Control^ and EVs^FAM3C^). **(C)** Representative images of lung sections stained for FAM3C (brown) and Ki67 (red) positivity. Original images were captured at 20x magnification; box showed cropped image of the indicated area prior to image size reduction.

Having demonstrated the effect of TDEs in inducing cell motility *in vitro*, we next explored their role in lung tumor colonization *in vivo*. To achieve this, we performed lung colonization studies using the tail vein injection model. Mice were randomly assigned to receive a 7-weeks' regimen of twice-weekly intravenous infusion of either PBS (negative control), EVs^Control^ or EVs^FAM3C^. As summarized in **Figure 4A,** the mice were first primed with TDEs for 2 weeks prior to a one-time tail vein injection of A549 cells mixed with either PBS, EVs^Control^ or EVs^FAM3C^. The animals continued to receive biweekly instillation of PBS or the appropriate EVs for another 5 weeks and then were sacrificed at the end of week 7. As characterized previously in **Figure 2E**, A549 cells steadily colonized the lung and formed large tumor nodules with open lacy appearance in all three treatment groups (**Figure 4B**). In this lung colonization model, the established A549 tumor nodules have the propensity to slowly seed new aggregates of carcinoma cells within the normal lung parenchyma (N) as seen in the PBS treated control group (**Figure 4B, upper left**). In comparison with mice exposed to PBS, animals treated with EVs^Control^ and EVs^FAM3C^ had respective ~2-fold and 4-fold increase in the number of tumor aggregates that appear as cell clusters with high pan-cadherin staining (**Figure 4B, bottom right**). This is concordant with recent reports on the role of TDEs in lung metastasis 18, 19, 28, that TDEs can communicate cell to cell metastatic cues. These lung tumor aggregates were found to express FAM3C (brown) and Ki67 positive cells (red) (**Figure 4C**), providing further evidence that FAM3C is an important mediator of carcinoma cell invasion and engraftment.

### FAM3C induces transcriptomic activation of oncogenic signaling

**Figure 5:**
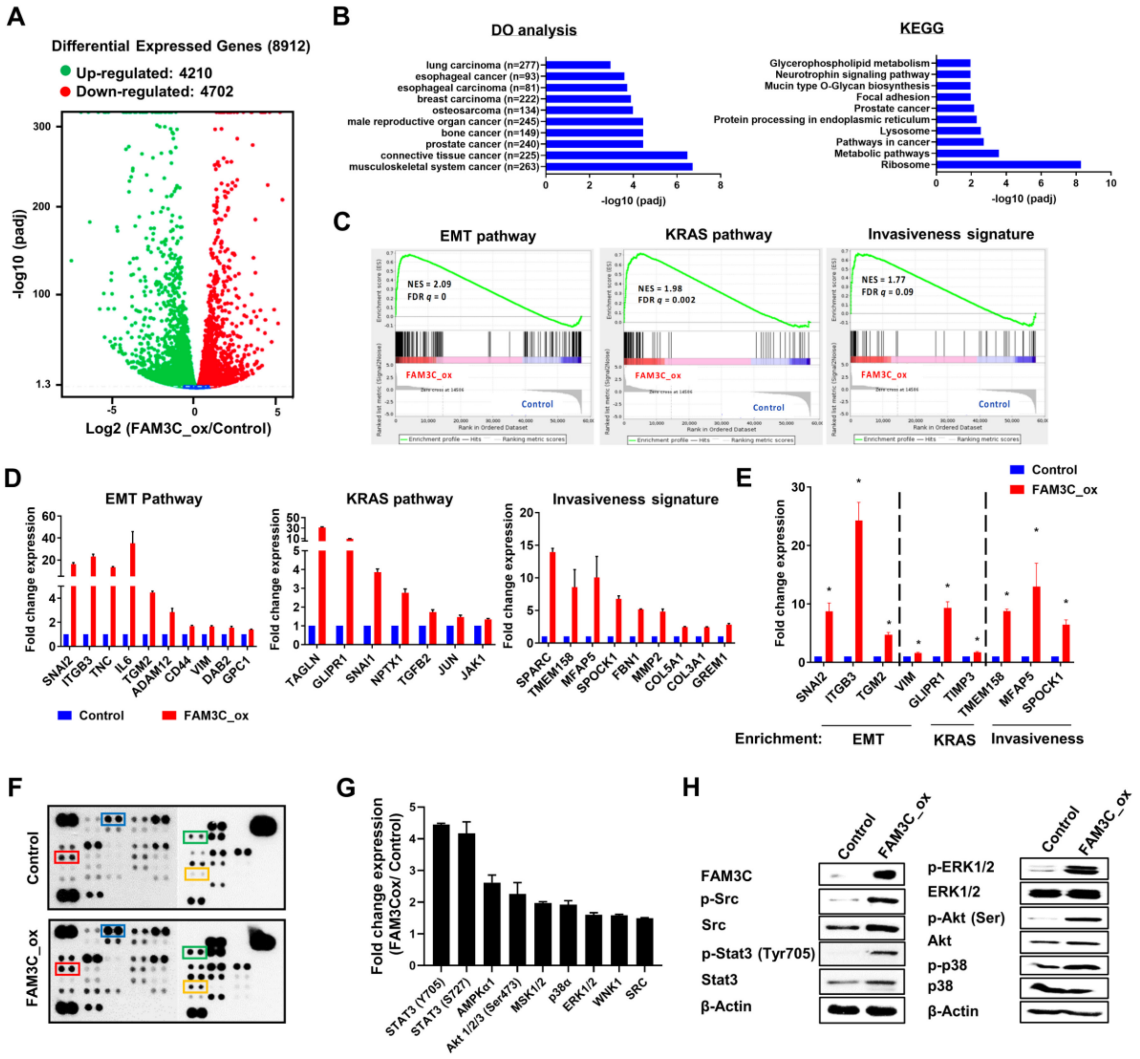
** Transcriptomic and molecular perturbations in FAM3C overexpressed cells.** RNA-Seq analysis of FAM3C_ox cells versus control cells. **(A)** Volcano plot of log_2_ (fold change) and -log_10_ (adjusted p-value) of all identified genes between control and FAM3C-overexpressing cells. **(B)** The top 10 enriched disease ontology (left panel) and KEGG pathway (right panel). **(C)** GSEA analysis of significantly enriched genes in FAM3C-overexpressing cells vs the control cells. NES, normalized enrichment score; FDR, false discovery rate. The red color scale indicates the positive and blue color indicates the negative correlation. **(D)** Histograms plotting the gene targets within the significantly enriched GSEA pathways. **(E)** Key targets genes from the RNA-Seq were validated using quantitative PCR (qPCR) analysis. **(F-H)** Kinome profiling of FAM3C_ox cells. **(F)** Human phospho-kinase array analysis was performed in H2170 and FAM3C_ox cells. Red box, blue box, green box and orange box indicates the phosphoproteins of Src, ERK1/2, Akt (T308) and STAT3 (Y705) respectively. **(G)** Bar graph represents the fold change expression of each phosphokinase proteins relative to control cells. **(H)** Western blot analysis of the indicated targets in H2170 and FAM3C_ox cells. Beta actin was used as a loading control.

We next sought to elucidate the molecular mechanisms underpinning FAM3C function. RNA-Seq analyses identified 4210 upregulated genes and 4702 downregulated genes in FAM3C_ox A549 cell lines compared to control cells (**Figure 5A**). Unsurprisingly, the transcriptomic perturbations evoked by FAM3C-overexpression were found to closely resemble curated gene signatures relevant to lung carcinoma, breast carcinoma, and prostate carcinoma, based on enrichment analyses using the Human Disease Ontology (DO) (**Figure 5B, left panel**). These differentially-expressed genes were also found to be overrepresented in KEGG-pathway ontologies related to focal adhesion and lysosome (**Figure 5B, right panel**), while gene-set enrichment analyses (GSEA) in the Molecular Signatures Database (MSigDB) further demonstrated significant overrepresentation of input genes in the hallmark gene set (identifier: M2572) for epithelial-to-mesenchymal transition (FDR *q*-value = 0), and two curated gene sets related to KRAS signaling (identifier: M2448; FDR *q*-value = 0.002) and tumor cell motility and invasiveness (identifier: M2572; FDR *q*-value = 0.09) (**Figure 5C-D**).

Selected genes involved in these GSEA pathways had been selected for independent validation using real-time quantitative PCR (**Figure 5E**). Notably, integrin beta 3 (*ITGB3*) was significantly elevated in FAM3C_ox cells. It has been previously reported that ITGB3 plays an important role in facilitating endocytosis-mediated uptake of EVs into recipient cells, and is critical for colonization of carcinoma cells in the lungs 19. The positive correlation of FAM3C and ITGB3 implicates a possible feedforward mechanism of FAM3C in favor of EVs uptake through promoting integrin internalization. Our transcriptomic analysis revealed that genes involved in the JAK-STAT3 pathway (*IL6*, *JAK1*) and KRAS signaling were upregulated in the FAM3C_ox cells (**Figure 5D-E**), thereby suggesting a possible involvement of FAM3C in signal transduction cascades. To examine this, we harnessed a human phosphokinase array to detect simultaneous perturbations in kinase activities. In H2170 cells, FAM3C overexpression were found to enhance the activation of oncogenic phosphoinositide-dependent signaling, as indicated by elevated levels of phosphorylated ERK1/2, MEK1/2, Akt, Src and Stat3 (Tyr705) (**Figure 5F-G**). These phosphorylation events were validated independently via immunoblotting (**Figure 5H**). Though these multi-omic analyses are meant to be hypothesis generating and nondefinitive, collectively the results suggest that FAM3C is involved in intracellular kinase signaling potentially through activation of Stat3 signaling.

### RalA is a functional interacting partner of FAM3C

Though FAM3C is known to induce metastasis, the signaling pathway mediating this is unclear. As FAM3C lacks a functional kinase domain, it is likely that interaction with other signaling molecules mediate this effect. Accordingly, we sought to identify potential interacting partners of FAM3C by employing the GPS-Prot bioinformatics database (GPS-Prot: Data Visualization for Protein-Protein Interactions (gpsprot.org)) (**Figure S10**). The Ras-related proto-oncogene A (RalA), a GTPase normally involved in regulating cell motility, vesicular trafficking, and endo/exocytosis 30-39, was identified as a plausible binding partner of FAM3C. Activation of RalA through phosphorylation at the C-terminal Ser194 35 residue is integral in Ras-mediated tumorigenesis, invasion, and metastasis 38, 39. We confirmed that these cell-biological effects are applicable to lung carcinoma, as overexpressing RalA in H2170 lung carcinoma cells increased Src and Stat3 phosphorylation (**Figure S7A**). In addition, RalA-overexpressing cells (RalA_ox) phenocopied those of FAM3C_ox with enhancement in their migration abilities, invasiveness, and *in vitro* malignant transformation (**Figure S7B-E**).

**Figure 6:**
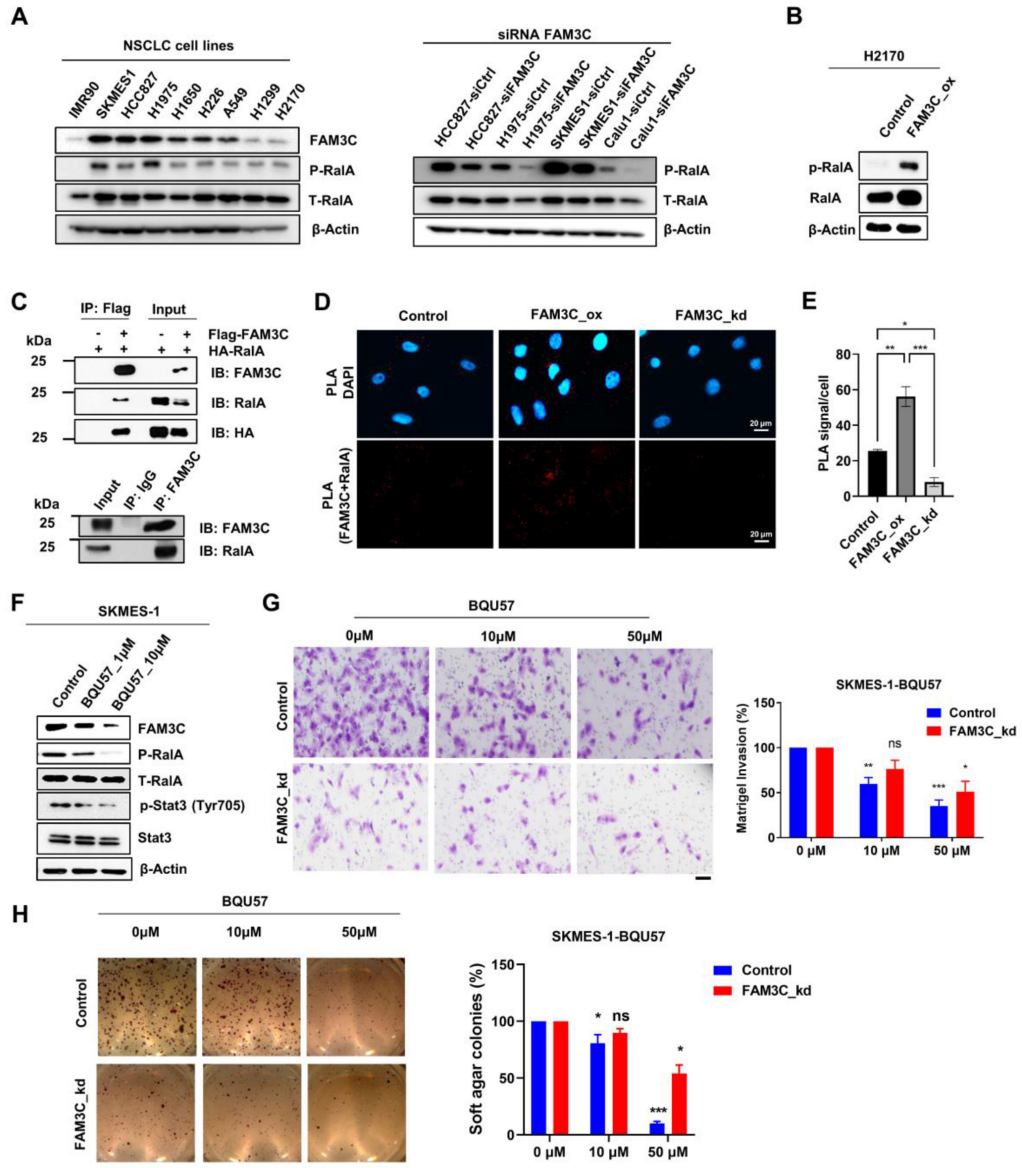
** RalA is an interacting partner of FAM3C. (A)** Differential expression of FAM3C and RalA in normal fibroblast IMR90 and NSCLC cell lines by Western blot (left panel). Western blot analysis of phosphorylated and total RalA expression in control and siRNA FAM3C knockdown NSCLC cells including HCC827, H1975, SKMES1 and Calu-1 (right panel). **(B)** Western blot analysis of phosphorylated and total RalA expression in control and FAM3C overexpressed H2170 cells. **(C)** Co-Immunoprecipitation (Co-IP) assay showing interaction of FAM3C and RalA. HEK293T cells were transfected with FLAG-tagged FAM3C and HA-tagged RalA plasmids. FLAG-FAM3C complex were pulled down by anti-FLAG beads and immunoblot with FAM3C and RalA antibodies (top panel). Immunoprecipitation of endogenous FAM3C protein was performed using anti-FAM3C antibody in SKMES-1 cells and immunoblot with FAM3C and RalA antibodies (bottom panel). **(D)** Microscopic images of FAM3C/ RalA staining by Duolink proximity ligation assay (PLA) in A549 cells. Red signals indicating proximity of FAM3C and RalA. DAPI was used to counterstain nucleus. **(E)** The PLA signals were quantified and expressed as number of signals per cell ± SEM (n > 3). **(F)** Effect of the RalA inhibitor BQU57 on protein expression of FAM3C and the phosphorylation of RalA and Stat3 in SKMES-1 FAM3C-knockdown cells. **(G, H)** Representative images are shown. Scale bar: 200 μM. Effect of BQU57 treatment on the anchorage-independent growth and invasion of SKMES-1 cells. SKMES-1 parental and FAM3C-knockdown cells were seeded in Matrigel invasion chambers **(G)** and soft agar **(H)** containing various concentrations of BQU57 drug with their corresponding bar graphs enumerating on the right panels. The bar graphs showed normalized data of BQU57 treatment to their corresponding controls. All data represented mean as ± SEM, ns: not significant, * *p* < 0.5, *p* < 0.01 and *** *p* < 0.001.

The notion that FAM3C is a novel *bona fide* binding partner of RalA was supported by multiple converging lines of evidence: First, we observed in immunoblot assays that FAM3C expression correlated with levels of total and especially phosphorylated RalA (Ser194) across an array of lung carcinoma cell lines (**Figure 6A, left**). This was further corroborated by the reciprocal observations that H2170 FAM3C_ox cells expressed higher p-RalA (**Figure 6B**), whereas FAM3C silencing in four FAM3C highly expressed lung cancer cell lines ablated expression of RalA phosphorylation (**Figure 6A, right**). Second, protein-protein interaction between FAM3C and RalA was confirmed by biochemical co-immunoprecipitation (Co-IP) assays through the overexpression of HA-tagged RalA and FLAG-tagged FAM3C either alone or simultaneously in HEK293T cells. As shown in **Figure 6C** (top panel), both HA and RalA were detected in the FLAG-FAM3C and HA-RalA co-transfected complex by immunoblot analysis after immunoprecipitation using anti-FLAG M2 magnetic beads. To dispel the notion that FAM3C interacts with RalA only at artificially high levels of protein expression, we repeated the experiment with FAM3C-high SKMES-1 lung carcinoma cells and confirmed the endogenous interaction of RalA and FAM3C (**Figure 6C**, bottom panel). Third, to further reinforce the findings from Co-IP assays, we also utilized an *in situ* Duolink proximity ligation assay (PLA) and confirmed the proximal co-localization of FAM3C with RalA in A549 parental and FAM3C_ox lung carcinoma cells (**Figure 6D-E**). In contrast, there was reduced PLA signals in the FAM3C_kd counterpart, suggesting a clear association between FAM3C and RalA. Collectively, the compatible findings provide consistent evidence that RalA is a functional interacting partner of oncogenic FAM3C.

We next explored strategies in targeting FAM3C-driven cancers. While there is no specific antagonist for FAM3C, we proposed to exploit RalA inhibition. By generating stable RalA-silenced A549 cells (**Figure S8A**), we showed that reduced expression of RalA could suppressed the oncogenicity associated with FAM3C (**Figure S8B-D**). Interestingly, the effect of recombinant FAM3C on cell migration and invasion was suppressed in shRalA cell line (**Figure S8C-D**), thereby confirming the dependency of FAM3C on RalA. Accordingly, we wondered if RalA inhibition may exert anti-tumor effect in FAM3C-driven cells by testing the efficacy of BQU57, a covalent small molecule inhibitor of RalA 32, 40. Immunoblotting analysis demonstrated that BQU57 dose-dependently reduced the expressions of both p-RalA and FAM3C with concomitant suppression of Stat3 phosphorylation, which is downstream effector of FAM3C (**Figure 6F**). Functionally, BQU57 exhibited dose-dependent inhibitory effects on the oncogenicity in SKMES-1 control cells, as demonstrated by the reduced cellular invasiveness (**Figure 6G**) and anchorage-independent growth (**Figure 6H**).

## Discussion

As a secreted protein, FAM3C is released into the extracellular milieu through unconventional mechanisms of protein secretion such as autophagy-dependent secretion 41 and plasminogen-urokinase plasminogen activator receptor-regulated secretion 42. Notwithstanding, the precise cell-biological and molecular crosstalk through which FAM3C acts has remained poorly defined. As FAM3C was found to exhibit an unexpected β-β-α fold 43 and also undergo covalent dimerization 44, it is believed to represent a structurally distinct and previously-unknown class of signaling molecules that is evolutionarily-unrelated 23 to classical four-helix cytokines. This motivated us to elucidate its role as a functional cargo molecule of carcinoma EVs. Several pertinent EVs mediated pathways have already been established in the metastatic cascade. Our current work adds to this metastatic paradigm in NSCLC, demonstrating that FAM3C carried in EVs of NSCLC overexpressing the protein promulgates distant colony formation at least in the lungs.

High expression of FAM3C is associated with poorer survival outcomes in multiple malignancies. This is explained by its role in promoting metastases; in pancreatic carcinoma for example, FAM3C is associated with hepatic metastases 17. Recently, its overexpression is reported to be regulated by copy number gains in carcinomas 25. Here, we present several lines of evidence to support the notion that FAM3C is prominently involved in colonization of lung carcinoma. First, comprehensive analyses on TCGA pan-cancer transcriptome revealed the high expression of *FAM3C* both in lung adenocarcinoma and squamous cell carcinoma. Second, distribution of *FAM3C* transcript is heterogenous across various cell types 26, and significant enrichments of *FAM3C*-positive populations are observed in lymph node and brain metastases of lung carcinoma patients. Third, protein-based analyses on tumor specimens and circulating EVs isolated from lung carcinoma donors detected an increasing level of FAM3C that is specifically associated with lung carcinoma progression. Fourth, we have demonstrated that delivery of FAM3C in lung carcinoma cells - either by ectopic gene expression or direct EVs uptake - significantly induced *in vitro* cell motility and *in vivo* lung colonization. Given these distinct lines of evidences, aberrant FAM3C expression is likely to represent a prognostic biomarker for predicting the risk of developing metastatic disease, and opens an opportunity to investigate RalA inhibitors as potential targeted therapies.

**Figure 7:**
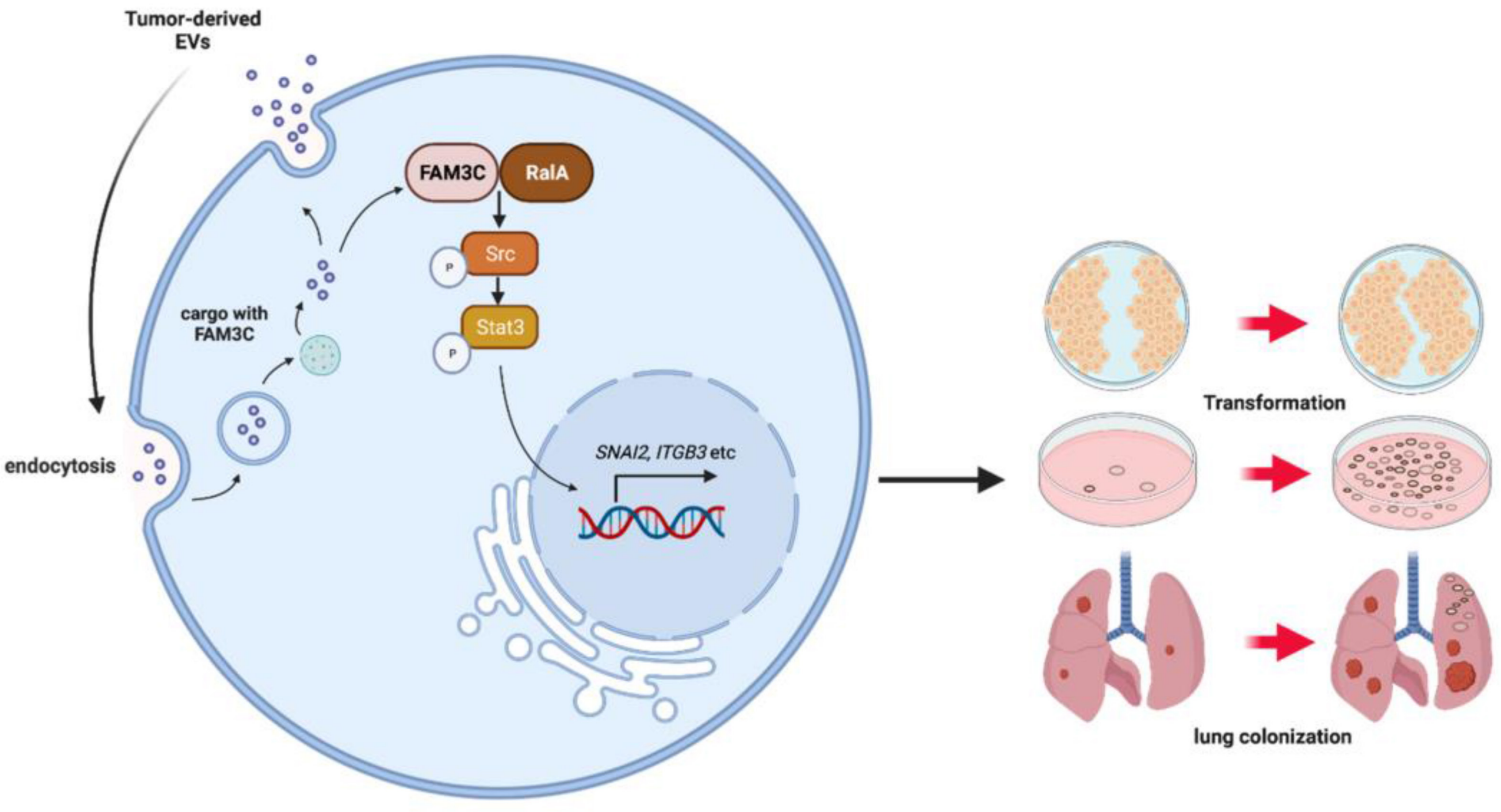
** Schematic diagram of the potential role of EVs-derived FAM3C in lung tumor invasion and distant colonization.** EVs-derived FAM3C activates the phosphorylation of RalA (Ser194) in lung carcinoma cells. RalA GTPase and subsequently activates the phosphorylation of SRC and STAT3 (Tyr705) in the downstream signaling pathway. This oncogenic activity induces cellular transformation and distant colonization in the recipient cells.

FAM3C appears to be overexpressed across NSCLC and is not associated with common driver oncogenes in lung carcinomas such as EGFR mutations. It is significant that FAM3C is present in both the primary tumor as well as the secretome as part of the circulatory EVs. This suggests that NSCLC utilizes vesicular FAM3C to prepare secondary or distant tissues for colonization. In this context, FAM3C overexpression was associated with expression of ITGB3, a known cell-cell and cell matrix adhesion molecule, raising the possibility that vesicular FAM3C may induce ITGB3 expression to promote uptake into recipient cells, thus forming the foundation for the establishment of distant colonies. Our tail vein mouse models provide strong evidence for this with development of higher density of lung colonization in mice primed with TDEs enriched with FAM3C. These experiments support the oncogenic role of FAM3C not only through mediating intracellular signaling events, but also in disseminating this stimulatory signal to distantcarcinoma cells through the vesicular cell communication circuit, which culminates in colony formation in distant tissues (**Figure 7**).

It is pertinent to unravel the cell signaling mechanisms contributory to this invasiveness phenotype since FAM3C has physiological functions in lipid and glucose metabolism 45, osteogenic differentiation 46, retinal laminar formation 47 and brain amyloid peptide formation 48. Consequently, direct inhibition of FAM3C may result in undesirable off-target effects. Though FAM3C can mediate carcinoma phenotype through various pathways including RAS, Jak/Stat3, and TGFβ, our work gives credence to its stimulation of migration and invasiveness through association with RalA, a Ras GTPase that is activated in many carcinomas, through downstream signaling pathways that include Src, JNK, NF-kB, cyclin D and Aurora A that may lead to cellular transformation. Despite so, it remains uncertain how RalA signaling is activated in carcinomas apart from its canonical upstream RalGEF effectors. As RalA co-precipitates with FAM3C, levels of both proteins are concordant, RalA silencing reduces FAM3C levels and abrogates FAM3C mediated cell migration, RalA signaling via direct interaction with FAM3C is likely, making RalA inhibition a strong candidate for disruption of TDE carried FAM3C effects.

The TGF-β signaling plays a prominent role in EMT and metastasis 49. While FAM3C acts as a downstream effector of TGFβ receptor 23, 24, it is notable that aberrant FAM3C expression is able to induce mesenchymal phenotype independently of TGF-β through the activation of MAPK/Erk pathway 22, 23. Given that the lack of correlation between FAM3C expression and its known receptor LIFR in lung cancer, it signifies the need to elucidate the underlying mechanisms of its signal transduction. As depicted in our model, cells incubated with TDEs isolated from FAM3C-overexpresssed cells are found to transduce pro-invasive cues, likely via a novel RalA-FAM3C axis that engender the downstream activation of MAPK and Src pathways. These observations recapitulate previous observations on the pro-metastatic cues of secreted FAM3C 50, and raise the possibility that EVs secreted from primary tumors are essential to the tumor-microenvironment interaction. RalA GTPase functions downstream of RAS as one of its oncogenic pathways and RalA is active in the GTP bound form. Recently, inhibitors of RalA have been developed including BQU57 that binds preferentially to the allosteric site of inactive RalA-GDP, preventing it from being activated and bind to its downstream signaling partner RalBP1. This inhibition of FAM3C by inhibition of RalA may avoid interrupting its physiological function and yet disrupting its gain of function oncogenicity.

While our *in vitro* findings show that uptake of FAM3C through EVs could regulate tumor localization, the mechanism of EV transmission remains unclear. The upregulation of ITGB3 transcript in FAM3C overexpressed cells suggests a direct impact on endocytosis. In addition, RalA regulates EVs processing through medication by exocyst complex proteins SEC5 and EXO84. In this context, inhibition of RalA would impair EVs trafficking and release, potentially impacting on vesicular FAM3C-mediated carcinoma cell invasiveness. Yet, this hypothesis is unexplored due to the limitation of our animal models that restricts the examination of intercellular transfer of FAM3C. As a follow up study, we intend to label TDEs with carboxyfluorescein succinimidyl ester (CFSE) for live tracking of TDE uptake within the tumor compartment. Further studies are warranted to explore the underlying mechanisms of RalA-FAM3C interaction, binding sites and protein stability, etc.

In summary, our current study describes a novel mechanism for FAM3C-driven carcinogenesis which is not relevant to expression level of LIFR. Though our study was pertinent in lung carcinoma, it may be relevant for other malignancies that highly express FAM3C, especially in EVs of cancer patients. It would be important to extend the investigation into these carcinomas to determine the role of FAM3C. Stratification of patients based on high FAM3C expression in plasma EVs may pave the path for developing strategies against widespread tumor colonization.

## Supplementary Material

Supplementary methods, figures and tables.
