# Supplementary material for: FAM3C in circulating tumor-derived extracellular vesicles promotes non-small cell lung cancer growth in secondary sites
Source: Theranostics. 2023 Jan 1;13(2):621–38. doi: 10.7150/thno.72297 (PMC9830426; doi:10.7150/thno.72297)
Supplement: Supplementary file 1 — Supplementary methods, figures and tables. [file thnov13p0621s1.pdf]

Supplementary Information

FAM3C in circulating tumor-derived extracellular vesicles promotes non-small cell lung cancer growth in secondary sites Win Lwin Thuya, Li Ren Kong, Nicholas L. Syn, Ding Ling Wen, Esther Sok Hwee Cheow, Regina Tong Xin Wong, Tingting Wang, Robby Miguel Wen-Jing Goh, Song Hongyan, Migara K. Jayasinghe, Minh TN Le, Hu Jian Cheng, Wei Peng Yong, Soo Chin Lee, Andrea Li-Ann Wong, Gautam Sethi, Ross A. Soo, Huynh The Hung, Paul Chi-Lui Ho, Jean-Paul Thiery, Siu Kwan Sze, Tiannan Guo, Henry Yang, Yaw Chyn Lim, Lingzhi Wang, Boon Cher Goh

List of Supplementary Material

1) Supplementary Methods

2) Supplementary Figures

- Figure S1, related to Figure 1
- Figure S2, related to Figure 1
- Figure S3, related to Figure 1
- Figure S4, related to Figure 2
- Figure S5, continuation of Figure 2
- Figure S6, related to Figure 3
- Figure S7, related to Figure 6
- Figure S8, related to Figure 6
- Figure S9
- Figure S10, related to Figure 6

3) Supplementary Tables

- Table S1: Top 20 of Log2 fold change EV proteins in Tumor cells versus normal fibroblast cells
- Table S2: The expression of FAM3C in NSCLC tissue array (n=100)

- Table S3: qPCR primers used in this study
- Table S4: shRNA sequences used in this study

1) Supplementary Methods

Cell Culture

Lung carcinoma cell lines utilized in this study were purchased from the American Type Culture Collection (ATCC) (Manassas, VA, USA); they include SKMES-1, HCC827, H1975, H1650, Calu-1, H2172, ChaGo-K, H520, H1299, H226, A549, and H2170 All the NSCLC cell-lines were maintained in RPMI-1640 medium (Nacalai Tesque, Kyoto, Japan) supplemented with 10% FBS, 1% L-glutamine and 1% antibiotics. Human normal lung-derived fibroblast IMR90 was maintained in MEM medium. Cell cultures were performed at 37°C in a humidified atmosphere of 5% CO2.

EVs isolation

Cells were cultured in T75 cell culture flasks until 80-90% confluency. The culture media were discarded and the cells were washed with 1x phosphate- buffered saline (PBS) at least 3 times to remove all bovine EVs. Then, the cells were cultured in serum-free RPMI-1640 or MEM supplemented with 1% L-glutamine and 1% penicillin/streptomycin. After 48 hours, serum-free conditioned culture media were collected and subjected to sequential centrifugation at 200 x g for 15 minutes followed by centrifugation at 2000 x g for 20 minutes at 4°C. These supernatants were passed through 0.2 μM filter. Next, the supernatants were concentrated to 3-5 mL using Amicon Ultra 15 mL 10K MWCO cellulose membrane (Millipore, Bedford, MA, USA) and ultracentrifuged at 110,000 x g for 2 hours at 4°C using a SW 55Ti rotor in a Beckman L100-XP Ultracentrifuge (Beckman Coulter, California, USA). Then EVs were further purified by 30% sucrose cushion ultracentrifugation at 110,000 x g for 2 hours at 4°C. The supernatants were removed, and the EV pellets were washed in 1x PBS and ultracentrifuged at 110,000 x g for 2 hours at 4°C and stored at -80°C prior to subsequent analyses.

Plasma-derived EVs isolation

Under approved protocol (Protocol #NS02/04/09), blood samples were obtained from 102 healthy subjects and 111 cancer patients. EVs isolation on individual patient plasma were isolated using Total Exosome Isolation Kit (from plasma) (Thermo Fisher Scientific, Cambridge, MA) according to the manufacturer's protocol, without

Proteinase K treatment. Harvested plasma-derived EVs was resuspended in 100 µL of 1 x PBS and stored at − 80 °C until sample processing.

In-gel digestion

The gels were manually excised according to protein bands and were subjected to reduction with 10 mM DTT (Sigma Aldrich, USA) in 25 mM ammonium bicarbonate (ABB) at 56°C for 60 minutes followed by alkylation with 55 mM iodoacetamide (IAA) in 25 mM ABB at room temperature in the dark for 45 minutes and dehydration with 50% acetonitrile in 25 mM ABB. The gel pieces were digested with sequencing grade modified trypsin (Promega, USA) in a protease:protein ratio of 1:100 at 37°C overnight. The trypsin digestion was repeated under the same condition for another 4 hours to facilitate complete digestion of proteins into peptides. The peptides were then extracted with 50% isopropanol and 5% acetic acid followed by 50% acetonitrile (ACN) and 5% formic acid (FA). The extracted peptides were dried in concentrator eppendorf vacuum and desalted using a Sep-Pak C18 cartridge (Waters, Milford, MA) as described below. The C18 cartridges were conditioned with 100% acetonitrile followed by equilibrating with 0.1% trifluoroacetic acid (TFA). Peptides dissolved in 0.1% TFA were loaded to the conditioned C18 cartridge, washed with 0.1% TFA and eluted with 75% acetonitrile and 0.1% trifluoroacetic acid. Then the peptides were dried in a centrifugal vacuum concentrator (Speed-vac system, Thermo).

Linear Trap Quadrupole Fourier Transform (LTQ-FT MS/MS) analysis

The peptide samples were dissolved in 20 µl of 5% ACN and 4% FA in HPLC grade water (J. T. Baker Inc, Philipsburg, New Jersey). The samples were then diluted with 100ul of HPLC grade water and centrifuged at 20,600g for 5 min to remove impurities. 100 µl of each sample were transferred to glass autosampler vials for LC-MS/MS analysis. The samples were analyzed using the LTQ-FT ultra mass spectrometer (Thermo Fisher Scientific, Inc., Waltham, MA) coupled with a ProminenceTM HPLC unit (Shimadzu), as previously described [63, 64] with some modifications. The peptide samples were injected from an auto-sampler (Shimadzu), concentrated in a Zorbax 300SB C18 column (5 mm x 0.3 mm, Agilent Technologies, Santa Clara, CA), followed by elution into an integrated nanopore column (75 µM x 100 mm, New Objective, Woburn, MA) packed with

C18 material (5 μM particle size, 300 A pore size; Michrom BioResources, Inc.). Mobile phrase A (0.1% FA) and mobile phrase B (0.1% FA, 100% ACN) were used to establish the 60-min gradient composed of 45 min of 8-35% B, 8 min of 35-50% B and 2 min of 80% B followed by re-equilibrating at 5% B for 5 min. The peptides were subsequently analyzed on LTQ-FT with an ADVANCE CaptiveSpray Source (Michrom BioResources) at an electrospray potential of 1.5 kV with auxiliary gas flow of 2, ion transfer tube temperature of 180°C, and collision gas pressure of 0.85 mTorr. The LTQ-FT was set to perform data acquisition in the data dependent mode as previously described [64]

Data analysis for LTQ-FT MS/MS

The raw data of LTQ-FT ultra was converted into the dta format using the extract_msn (version 4.0) program found in Bioworks Browser 3.3 (Thermo Electron, Bremen, Germany). The dta files were then converted to Mascot generic file format using an in-house program. Intensity values and fragment ion m/z ratios were not manipulated. The 'target' (forward IPI human sequences of IPI human protein database, version 3.34, 67758 sequences) and 'decoy' (reverse IPI human sequences) database were combined for the search using an in-house Mascot server (version 2.2.04, Matrix Science, Boston, MA) with precursor mass tolerance of 20 ppm and MS/MS tolerance of 0.8 Da. A maximum of 2 missed trypsin cleavage sites and 13C of 2 were allowed. Carbamidomethylation at Cys residues was set as a fixed modification, whereas oxidation at Met residues was set as variable modifications. Only proteins with a MOWSE score higher than 20, corresponding to $p < 0.05$ were considered and protein/peptide lists were processed and exported to Microsoft Excel using in- house script, for further analysis. The proteins identified in this study were classified according to their biological processes and molecular functions using online database STRING database Version 11 (https://string-db.org).

Scanning Electron Microscopy (SEM)

EV samples were first fixed in 2.5% Glutaraldehyde (Electron Microscopy Services, Hatfield, PA, USA) in 0.1M Sodium Cacodylate buffer, pH 7.4 (Electron Microscopy Services, Hatfield, PA, USA) for 60 min, and rinsed in 0.1M Sodium Cacodylate buffer (3 x 10 min) at 25°C. Samples were post-fixed in 1% osmium tetroxide (Electron Microscopy Services, Hatfield, PA, USA) in 0.1M Sodium Cacodylate buffer for 60 min in the dark and washed

in distilled water (3 x 5 min) at 25°C. Samples were dehydrated in ethanol of sequential concentrations (25%, 50%, 75%, 95% and 100% ethanol) for 10 min each at 25°C. The dried samples were than mounted with carbon tape and carbon conductive paint and observed under SEM (JSM-6330F, JEOL, Tokyo, Japan).

Western blotting

Cells were lysed in PIERCE™ RIPA lysis buffer, supplemented with Halt™ protease and phosphatase inhibitor cocktail (Thermo Fisher Scientific). The optical density of EV protein concentration was measured at 562 nm using bicinchoninic acid (BCA) Protein assay kit (Thermo Scientific, USA) and Infinite® M1000 Microplate Reader (TECAN, Switzerland). The proteins were separated on 8-12% SDS-PAGE gels and the electrophoresed gels were transferred into PVDF membranes. Nonspecific protein binding was blocked by incubating with 5% Non-fat milk (BioRad Laboratories, USA) in 0.1% PBS-Tween (PBST) at room temperature for 60 min. After blocking, membranes were incubated with primary antibodies at 4°C overnight followed by the appropriate species-specific Horseradish peroxidase-conjugated secondary antibodies at room temperature for 1 hour and detected using an enhanced chemiluminescence kit (GE Healthcare, USA). Blotting was performed with the following primary antibodies: E-cadherin, Alix, RalBP1, p-ERK1/2, total ERK1/2, p-MEK1/2, total MEK1/2, p-Akt, total Akt, p-p38, total p38, p-Src, total Src, p-STAT3 (Tyr705), total STAT3, beta actin and HRP-conjugated anti-Rabbit and anti-Mouse secondary antibodies from Cell Signaling Technology, USA; FAM3C, CD63 and total RalA from Abcam, UK; p-RalA (Ser194) from Merck Millipore; Vimentin from BD Biosciences, UK; LIFR, Tsg101 and CD9, from Santa Cruz Biotechnology, USA.

Immunohistochemistry (IHC) staining and scoring

The NSCLC tissue microarray comprising 45 cases of NSCLC tissues, 47 cases of tumor adjacent normal tissues and 8 cases of normal lung tissues was obtained from US Biomax, Inc. The TMA samples were manually stained using optimally diluted (1:400) anti-FAM3C antibody (Rabbit pAb, AbCAM). The slides were visualized by colour development using DAB chromogen from DAKO. Tissue sections were counterstained with hematoxylin, dehydrated and mounted. The staining of mouse lung sections with anti-pancytokeratin (Mouse mAb, clone AE1/3, AbCAM) was carried out on the Leica BondMax autostainer using standard protocol J (for Alkaline

Phosphatase, red). The double staining of the mouse lung sections with anti- human FAM3C (Rabbit mAb, Clone D1S2D XP, Cell Signaling) and anti-Ki67 antigen (Mouse mAb, clone MIB-1, Dako) was carried out using protocol F (primary antibody – 30 min incubation, for Horseradish Peroxidase, brown) and Protocol J (primary antibody – 15 min incubation, for Alkaline Phosphatase, red) respectively. Heat-induced epitope retrieval (HIER) with TRIS-EDTA pH9 (ER2 buffer) for 20 min was used to unmask the epitopes for cytokeratin and FAM3C; and HIER with Citrate buffer pH6 (ER1) for 20min was used for unmasking the Ki67 antigen.

The scoring criteria for FAM3C expression for the human lung tissue TMA were 0 for no expression, 1 for weak expression, 2 for moderate expression and 3 for strong expression in the tumor cells. For the quantification of tumor nodules, the number of tumor nodules (visualized by strong pan-cytokeratin staining) on 2 consecutive sections (100 µm apart) were assessed by 2 independent members of the group. The mean number of nodules per section per mouse was determined. The number of tumor aggregates in each FAM3C-stained section were counted by 2 independent assessors and the mean number of tumor aggregates per section determined. The criteria of a tumor aggregates are defined as (1) the single or small cluster of cells were cytokeratin or FAM3C positive and (2) each cluster is located in the normal lung parenchyma and at least 50 µm away from any established tumor nodules. Thus, newly proliferated cells at the invasive front of existing tumor nodules were excluded.

FAM3C plasmid constructs and lentiviruses transduction

Human FAM3C MISSION shRNA Lentiviral Transduction Particles and MISSION TRC2 Control Vector pLKO-puro TurboGFP shRNA were obtained from Sigma. The sequence for FAM3C shRNA was CCGGCTTGGTGTGTGCATGAGTATTCTCGAGAATACTCATGCACACACCAAGTTTTTG. The sequences for RalA and LIFR shRNA lentiviral vectors were listed in Supplementary Table 3. SKMES-1 and A549 cells (7 × 104) were seeded onto 6-well plates and transduced with FAM3C, RalA and LIFR shRNA and TurboGFP shRNA. The transduced cells were selected in RPMI 1640 medium containing puromycin at 1 µg/mL 48 h post-transfection. To generate FAM3C expression vectors, FAM3C Human Tagged ORF clone with pCMV6-AC-GFP vector was obtained from Origene. The complementary DNA expression plasmids was verified using direct sequencing at the 1st BASE DNA sequencing services.

In vitro invasion assay

Cell invasion capability was performed using a Corning® BioCoat™ Matrigel Invasion Chambers (#354480). Briefly, 750 µl of RPMI 1640 medium supplemented with 1% L-glutamine, 1% antibiotics and 10% FBS (chemoattractant) was placed in the lower compartment of each chamber. In the prewarmed and rehydrated upper chambers, 25,000-50,000 cells in 500 µl of RPMI 1640 medium without FBS were added, and the cells were allowed to migrate through the intermediate membrane for 24 hours at 37°C. Cells that invaded through the membrane were fixed in 100% methanol for 15 min and stained with 0.5% gentian violet (ICM, Singapore) for 45 min before being observed under Olympus CKX41 inverted microscope (Olympus Corporation, Tokyo, Japan).

Anchorage-dependent and -independent colony formation assays

For anchorage-dependent assay, human lung carcinoma (SKMES-1, A549 and H2170) control, FAM3C-overexpressed and shFAM3C-knockdown cells were seeded at a density of 5,000 cells/well in 6 well plates. After 7-14 days incubation at 37°C, cells were washed with 1x PBS, stained with 0.5% gentian violet (ICM, Singapore) for 30 min, washed with sterile distilled water and then photographed. For anchorage-independent assay, soft agar assays were performed. The human lung carcinoma (SKMES-1, A459 and H2170) control, FAM3C-overexpressed and shFAM3C knockdown cells (5000-10,000 cells/well) suspended in culture media containing 0.36% agarose were plated on the bottom layer of 0.6% agarose in culture medium covered by a top layer of complete media. Weekly, a fresh 300 µl of complete media was added to maintain humidity. Finally, 30 µl of MTT solutions (Promega, #G4000) were added into each well and incubated at 37°C for 2-3 hours on the final day of assay, approximately after 4 weeks of culture.

Extracellular vesicles uptake by NSCLC cells visualized with fluorescent labeling

The fluorescent reagent Exo-Green (System Biosciences) was utilized to label the protein component of H2170 lung carcinoma cell-derived EVs. EVs isolated from FAM3C-overexpressing and control cells were resuspended in 500 µl of 1x PBS. Next, Exo- Green reagent was added to the solution and incubated for 30 mins at 37°C and then the labelled EVs were washed with 1x PBS and re-pelleted using ExoQuick-TC EVs precipitation solution.

Exo-Green labelled control and FAM3C-overexpressed cells-derived EVs were incubated with H2170 parental cells in a 6-well plate for at least 2hr at 37°C. Fluorescent and bright field images were acquired with a ZEISS Axio Vert.A1 fluorescence microscope.

Proteinase K treated EVs

Isolated EVs pellets were re-suspended in 1x PBS and subsequently divided into three equal aliquots. Proteinase K enzyme treatment was performed with 0.1 mg/mL proteinase K with or without 1% Triton X-100 for 30 mins at 37 °C. One of the aliquots was treated without both proteinase K and Triton X-100 as control. Protease and phosphatase inhibitor was added to control the enzymatic activity of proteinase K. Then EVs were ultracentrifuged at 110,000 x g for 1 hours at 4°C and EVs pellets were lysed in RIPA buffer with protease and phosphatase inhibitor. EVs proteins were mixed with laemmi buffer and heated at 95° for 10 minutes.

2) Supplementary Figures

Figure S1, related to Figure 1

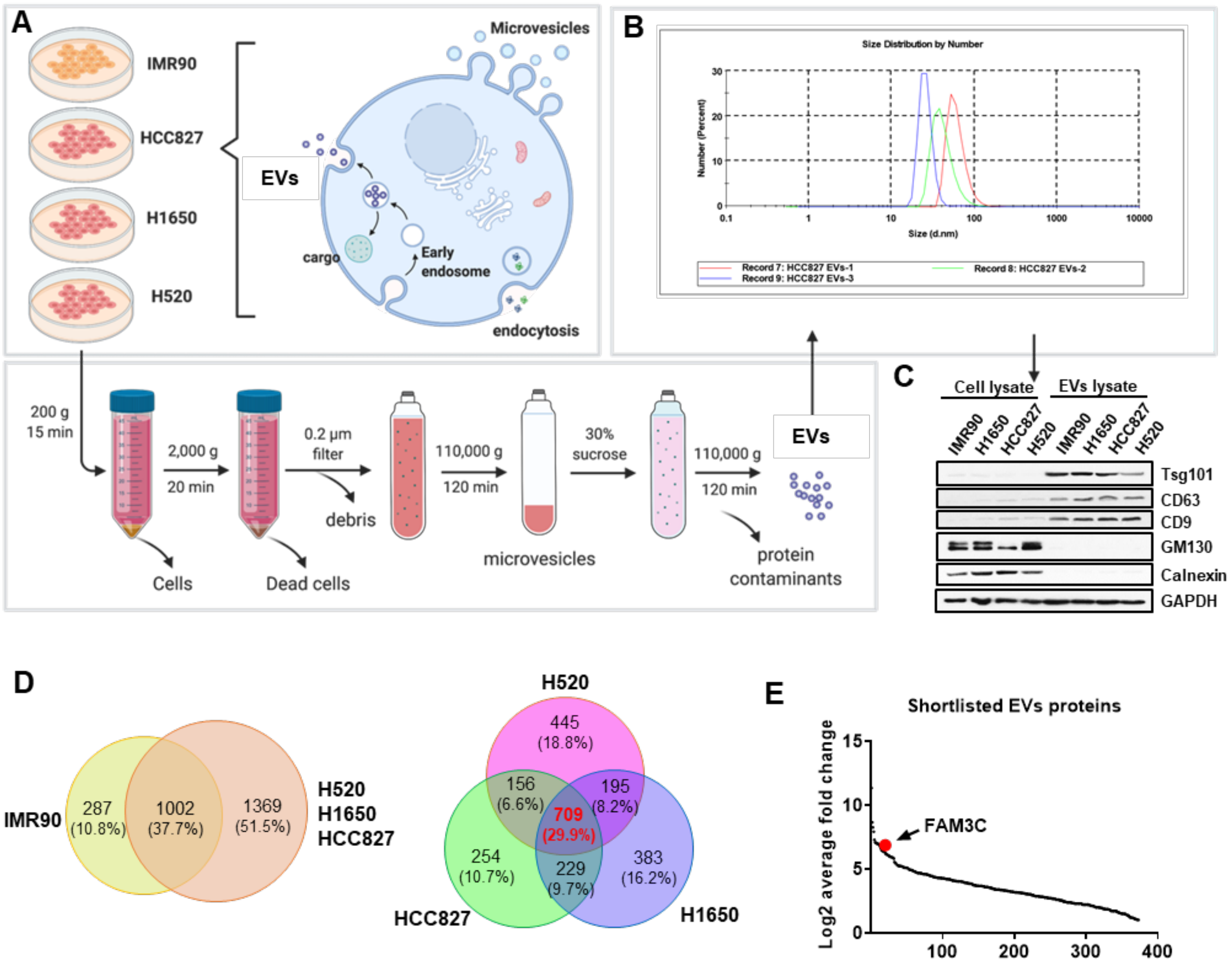

Figure S1. Proteomics analysis of tumour-derived EVs and validation of shortlisted FAM3C protein in human plasma. (A) Schematic illustration of the isolation and purification of cell lines derived EVs. EVs were harvested from cell culture supernatant of three lung cancer cells (HCC827, H1650 and H520) and one normal fibroblast cells, IMR90. Extracted EVs were isolated by differential centrifugation followed by ultracentrifugation. **(B)** Size distribution of HCC827 cell-derived EVs was assessed by the Zetasizer Nano ZS90. **(C)** Western blot analysis of exosomal markers (Tsg101, CD63 and CD9), golgi protein marker (GM130) and endoplasmic reticulum marker (Calnexin) in cell lysates and EVs isolated from the culture media of the respective cell lines. **(D)** Quantitative data analysis was performed using bioinformatics tools. Overlap of exosomal proteins from three lung cancer cell lines and normal fibroblast cell line (left panel). 709 proteins were found to be common among the three lung cancer cell lines (right panel). **(E)** Based on bioinformatics analaysis, 374 proteins were significantly upregulated by log2 fold change of tumour versus normal cells.

Figure S2, related to Figure 1

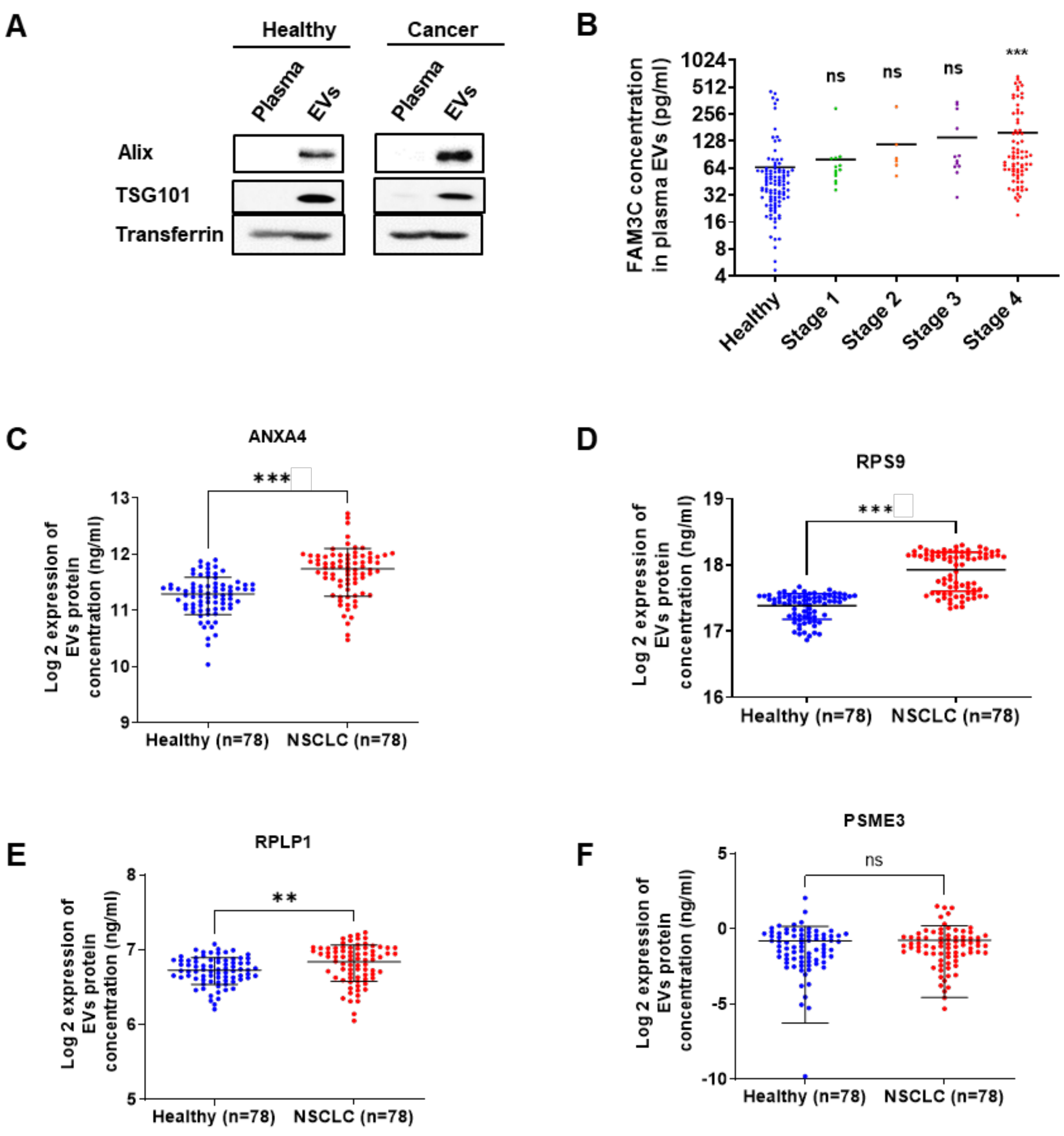

Figure S2. Validation of EVs candidate proteins in human plasma. (A) Western blot analysis of plasma EVs derived from healthy subject and NSCLC patient revealed that EVs markers Alix and Tsg101 were enriched in plasma EVs relative to plasma proteins. **(B)** Comparison of plasma EVs FAM3C concentrations between healthy subjects and NSCLC patients with different TNM subgroups. EVs proteins including ANXA4 **(C)**, RPS9 **(D)**, RPLP1 **(E)** and PSME3 **(F)** were validated by ELISA assay in plasma derived EVs samples from healthy control individuals (n=78) and NSCLC patients (n=78). Data were shown as scatter plots showing individual replicates and error bars indicate +/- SEM. ns: not significant, ** $p < 0.01$ and *** $p < 0.001$.

Figure S3, related to Figure 1

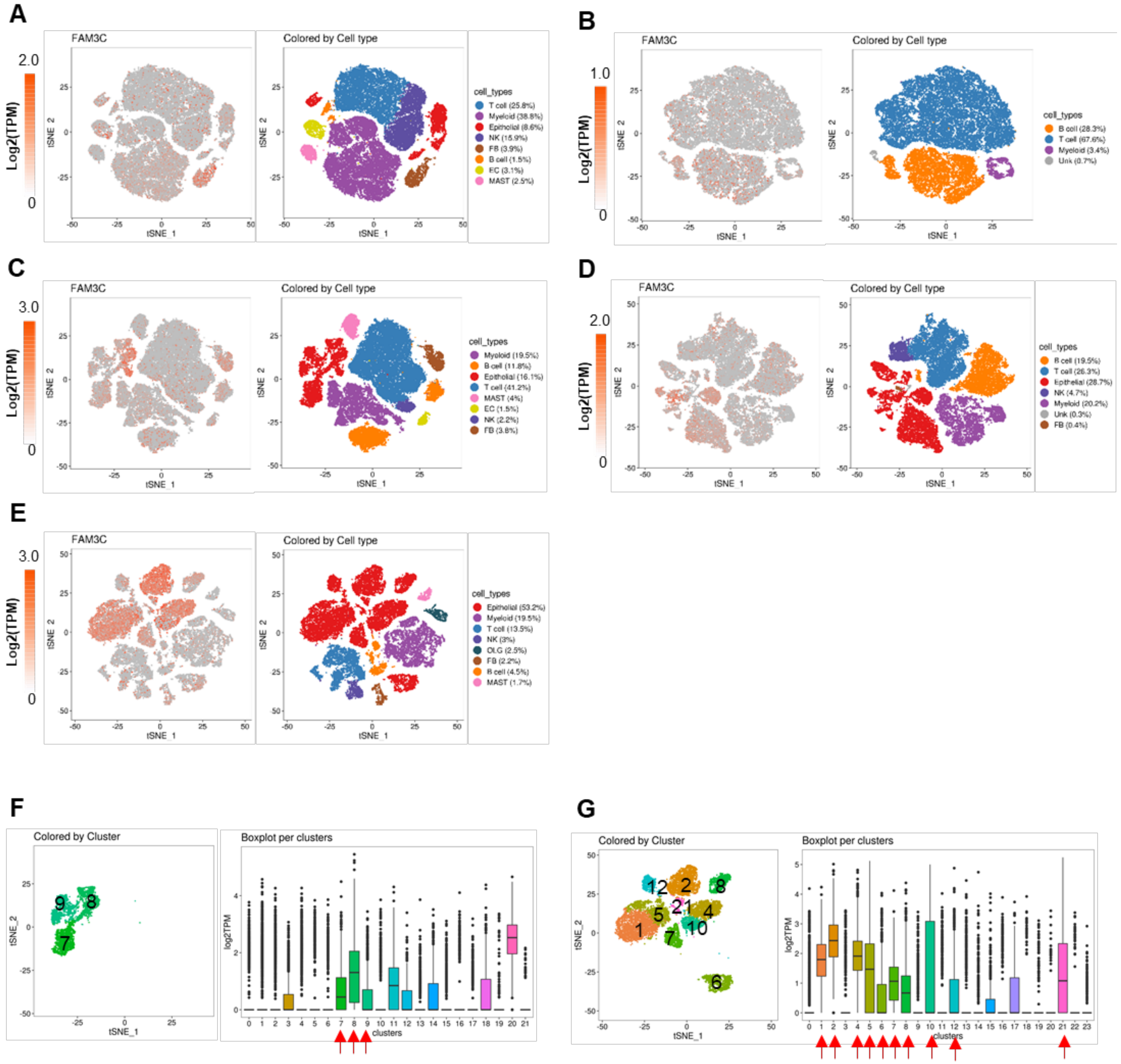

Figure S3. **Identification of FAM3C in normal lung tissues and tumors.** Single cell RNA sequencing (scRNA-Seq) analysis of 44 treatment-naïve lung adenocarcinoma (LUAD) cases, covering matched normal **(A)**, normal lymph node **(B)**, primary tumor **(C)**, metastatic lymph node **(D)** and brain metastases **(E)**. Right, tSNE plots to visualize cell-type clusters based of the known marker genes; Left, expression levels of FAM3C. Expression data is presented as log2 TPM (Transcripts Per Kilobase Million). NK, natural killer cells; FB, fibroblasts; EC, endothelial cells. Epithelial cell clusters of primary tumors **(F)** and brain metastases **(G)** were highlighted, with boxplot showing the log2 TPM values of FAM3C expression. Red arrows: epithelial cell clusters. Figures are generated using the public dataset provided by URECA Research Network (http://ureca-singlecell.kr).

Figure S4, related to Figure 2

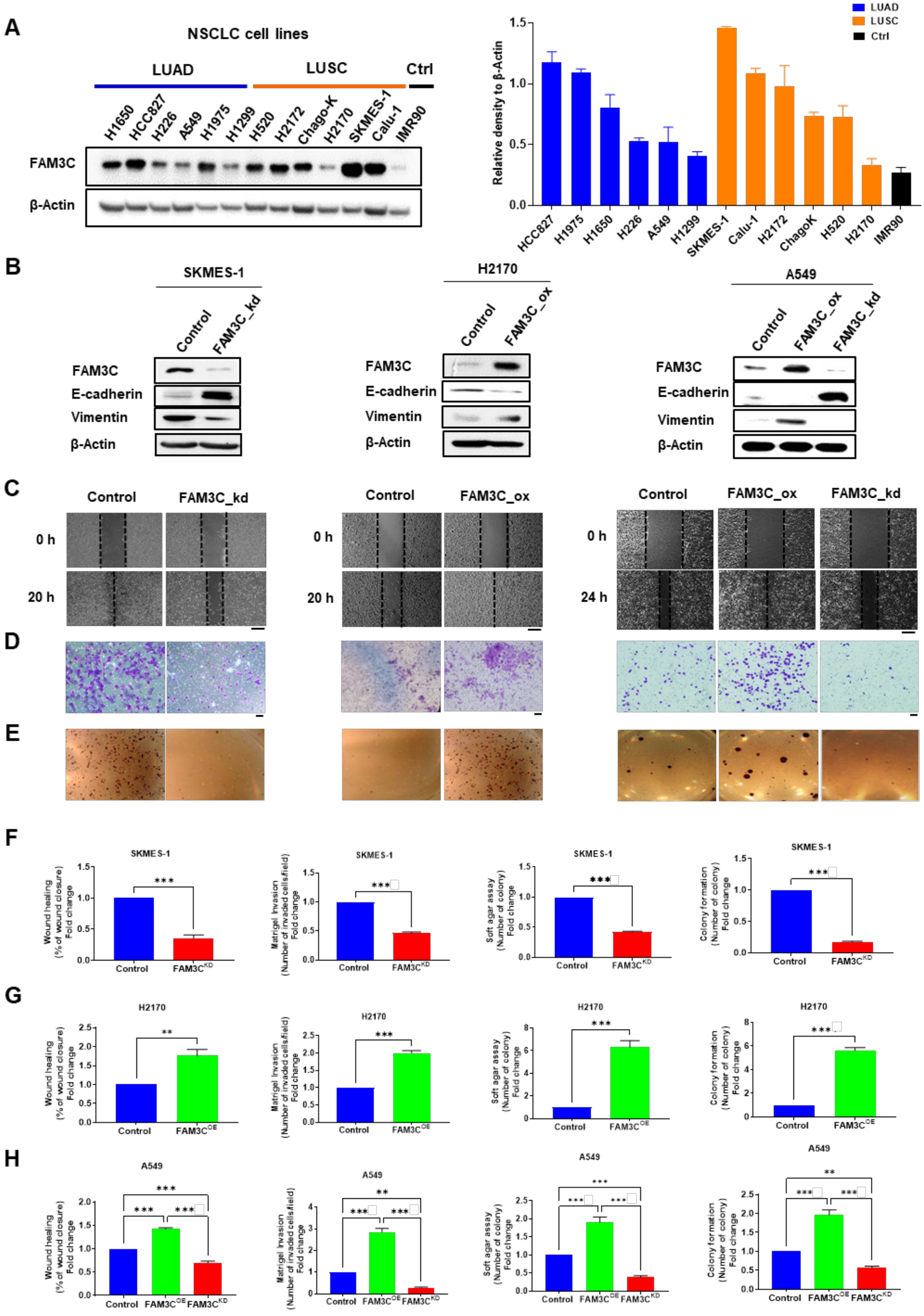

Figure S4. FAM3C increases EMT and invasiveness of lung carcinoma cells *in vitro*. Stable FAM3C-knockdown SKMES-1 and A549 cells were generated by lentivirus infection. Stable FAM3C overexpression H2170 and A549 cells were generated through plasmid transfection. **(A)** Western blot analysis of FAM3C in 12 NSCLC cell lines compared to normal lung fibroblast (NLF) cell line (IMR90). The histogram represents expression levels of FAM3C in normal lung fibroblast (black), LUAD (blue) and LUSC (orange) after normalization against β-actin. **(B)** The expression of E-cadherin and Vimentin in FAM3C-depleted and overexpressing cells was determined by using Western blot analysis **(C)** Representative images are shown. Scale bar: 200 μM. The effect of FAM3C knockdown and overexpression on cell migration was examined by wound healing assay. **(D)** Representative images are shown. Scale bar: 200 μM. The cell invasion ability of FAM3C knockdown and overexpressed cells was examined by Matrigel invasion assay. **(E)** Anchorage-independent growth was determined by colony formation in soft agar. **(F-H)** The corresponding bar graph enumerated wound healing, Matrigel-invasion, anchorage-dependent and anchorage-independent assays of the respective lung carcinoma cell lines. The data presented in all panels of this figure are the results of three independent experiments and are represented as ± SEM. ** $p < 0.01$ and *** $p < 0.001$.

Figure S5, continuation of Figure 2

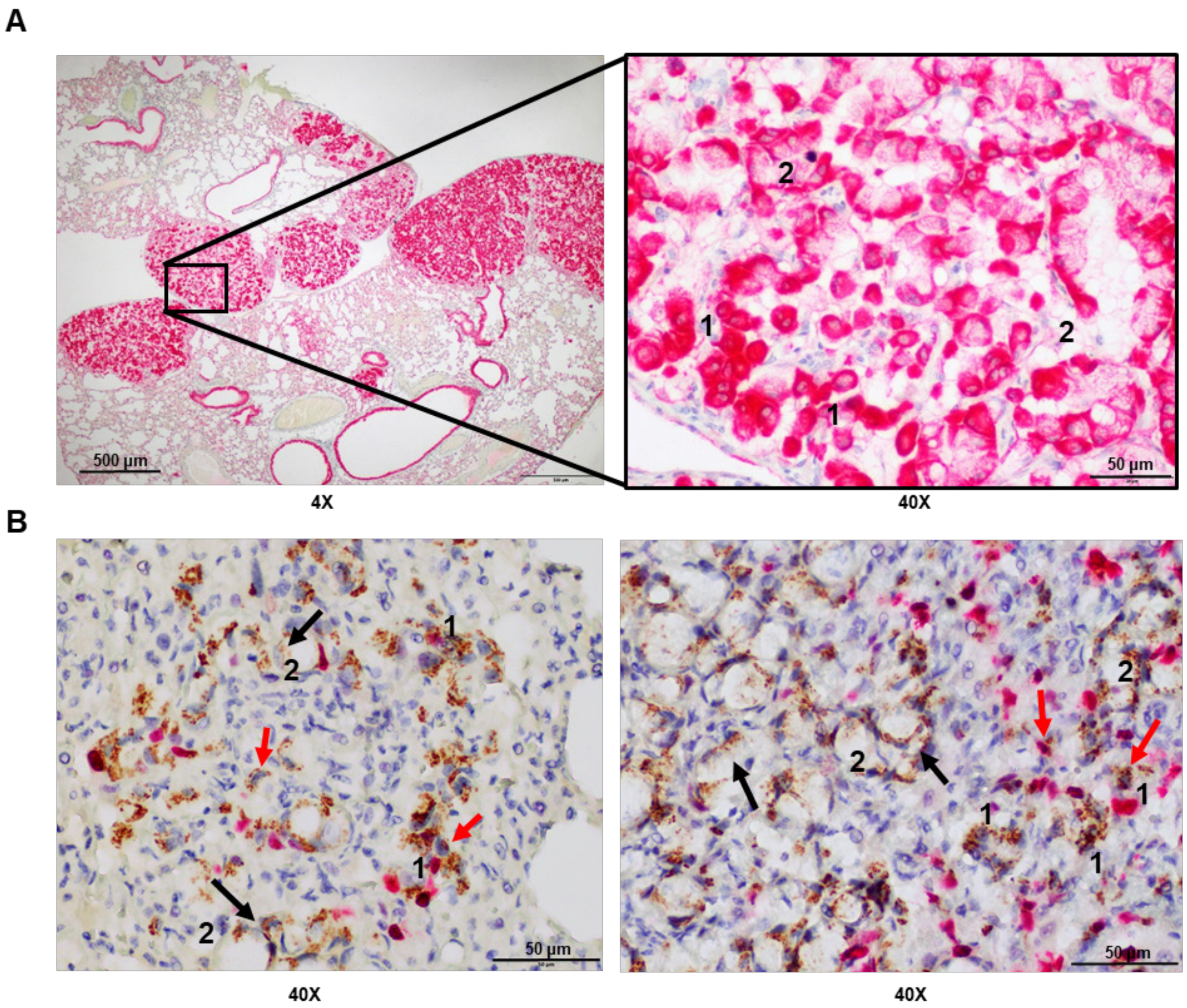

Figure S5. FAM3C impacts the invasiveness of lung carcinoma cells. (A) Representative images at 4x and 40x magnification of a FAM3C_kd tumor nodule stained with pan-cytokeratin (red). **(B)** Representative images of lung sections of A549 parental (left) and FAM3C_ox (right) tumors stained with FAM3C (brown) and Ki-67 (red) at 40x magnification.

Figure S6, related to Figure 3

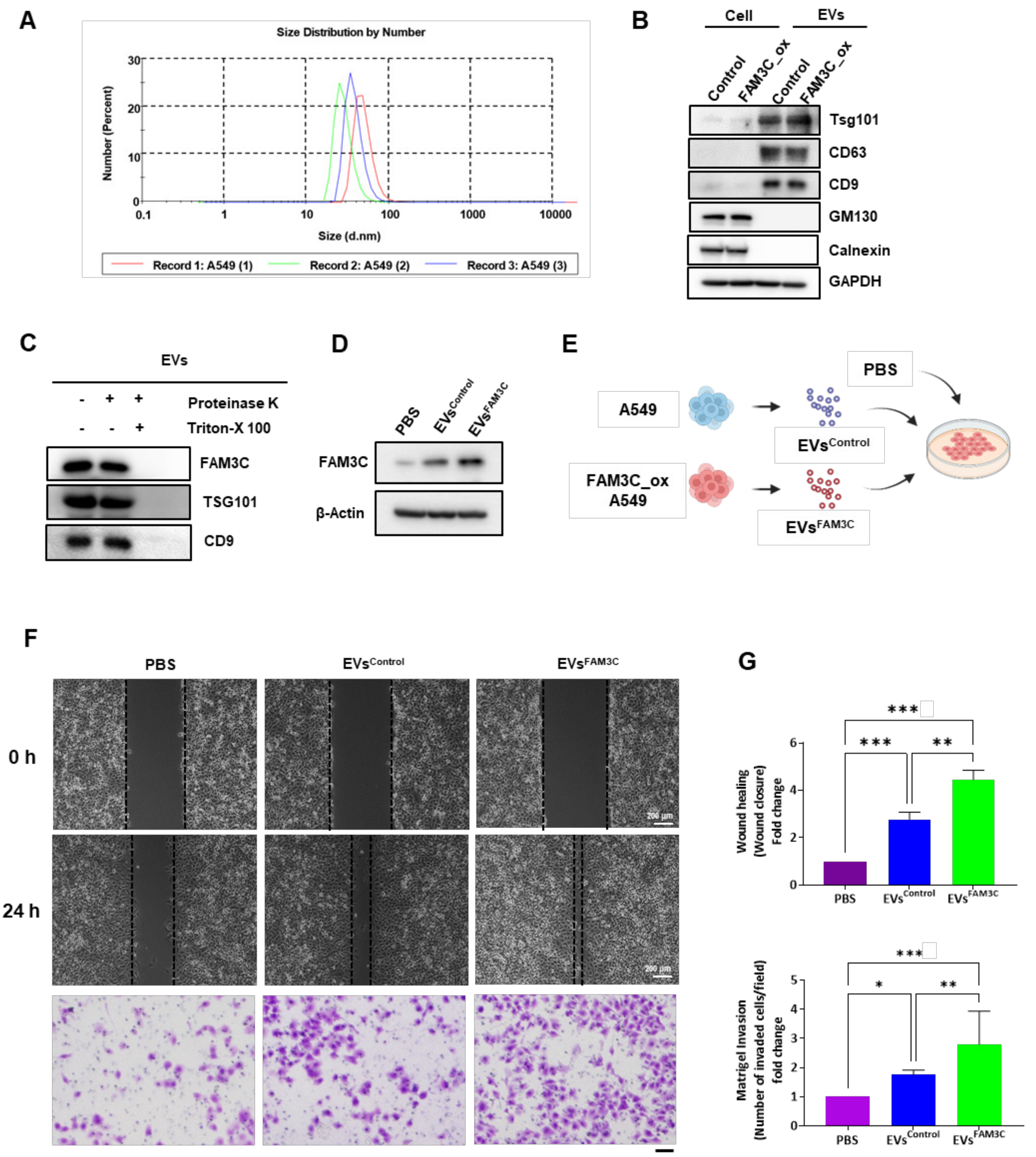

Figure S6. Tumor-derived EVs containing FAM3C promote invasiveness of lung carcinoma cells *in vitro*. **(A)** Size distribution of A549 cell derived EVs was assessed by the Zetasizer Nano ZS90. **(B)** Western blot

analysis of three positive (+) EVs markers (Alix, Tsg101 and CD63), and two EVs negative (-) markers (GM130 and Calnexin) in cell lysates and EVs isolated from the culture media of the respective cell lines. GAPDH served as loading control. **(C)** Proteinase protection assay to show stability of EVs. Western blotting analysis of A549 EVs treated with and without proteinase K and Triton-X 100. **(D)** Western blot analysis of FAM3C expression in A549 cells treated with PBS, 25 µg/mL of $EVs^{Control}$ and 25 µg/mL of EVs^{FAM3C}. **(E)** Experimental set up for EVs uptake by lung carcinoma cells. The EVs isolated from A549 control ($EVs^{Control}$) and FAM3C-overexpressed (EVs^{FAM3C}) cells were added into culture medium of parental A549 cells to evaluate for metastatic potential. **(F-G)** Effects of PBS, $EVs^{Control}$ or EVs^{FAM3C} are assessed with **(F)** wound healing (top) and Matrigel invasion assays (bottom) in A549 cells after treatment with 25 µg/mL of respective EVs. Representative images are shown. Scale bar: 200 µM. **(G)** Data are presented as mean of three independent experiments ± SEM. * $p < 0.05$, ** $p < 0.01$ and *** $p < 0.001$.

Figure S7, related to Figure 6

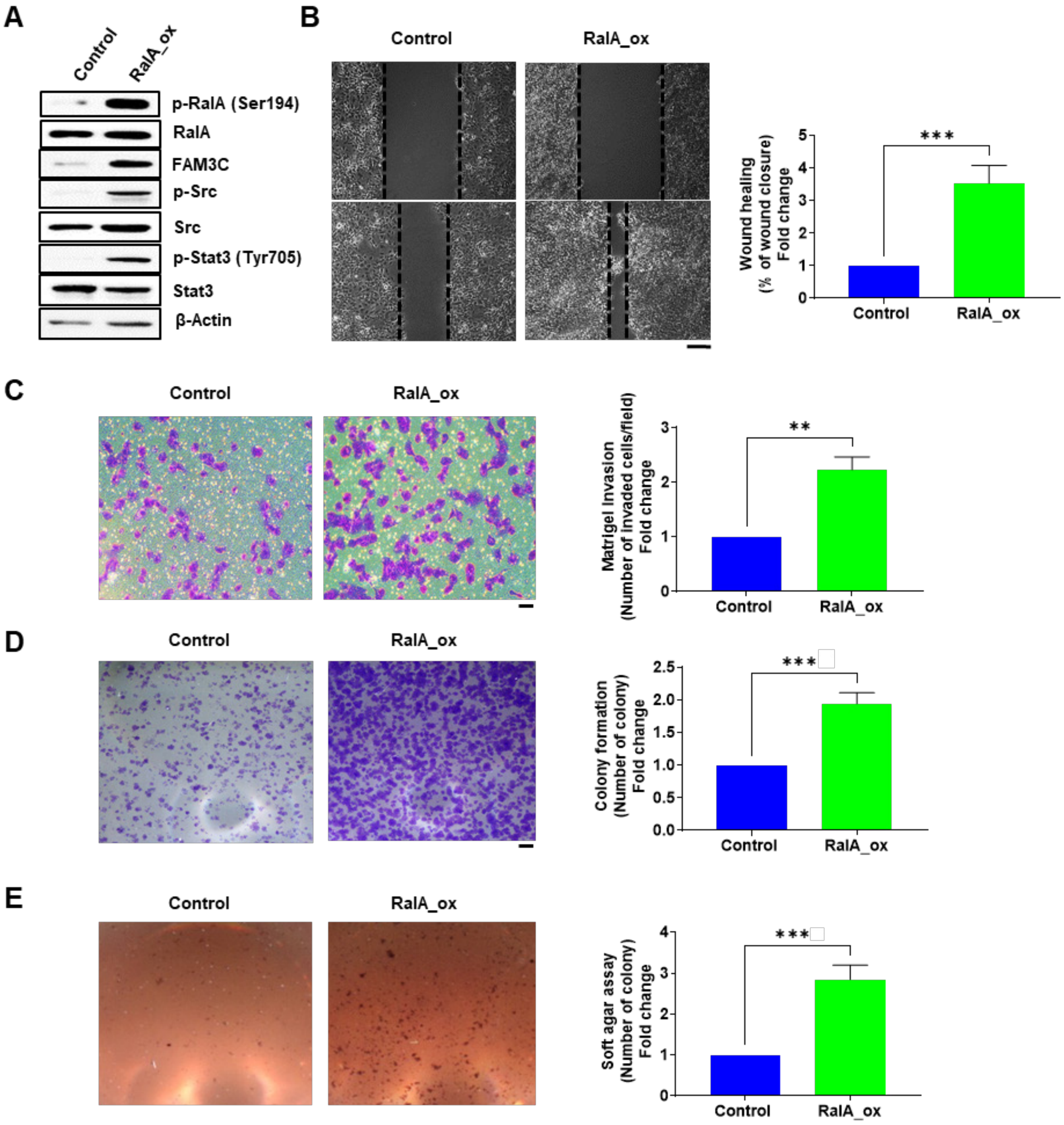

Figure S7. RalA overexpression increases cell motility and colony formation in H2170 lung carcinoma cells. Stable RalA overexpression H2170 cell line was generated through plasmid transfection. **(A)** Overexpression of RalA upregulates FAM3C and activates its downstream signaling pathway. **(B-C)** Representative images are shown. Scale bar: 200 µM. The cell motility of FAM3C overexpressed cells was examined by wound healing **(B)** and matrigel-coated transwell assay **(C)**. **(D-E)** Representative images are shown. Scale bar: 200 µM. Anchorage-dependent colony forming assay **(D)** and soft agar colony forming assay **(E)** upon RalA overexpression in H2170 lung carcinoma cells. The data presented in right panels of **(B-E)** are the results of three independent experiments. Data presented as mean ± SEM, ** $p < 0.01$ and *** $p < 0.001$.

Figure S8, related to Figure 6

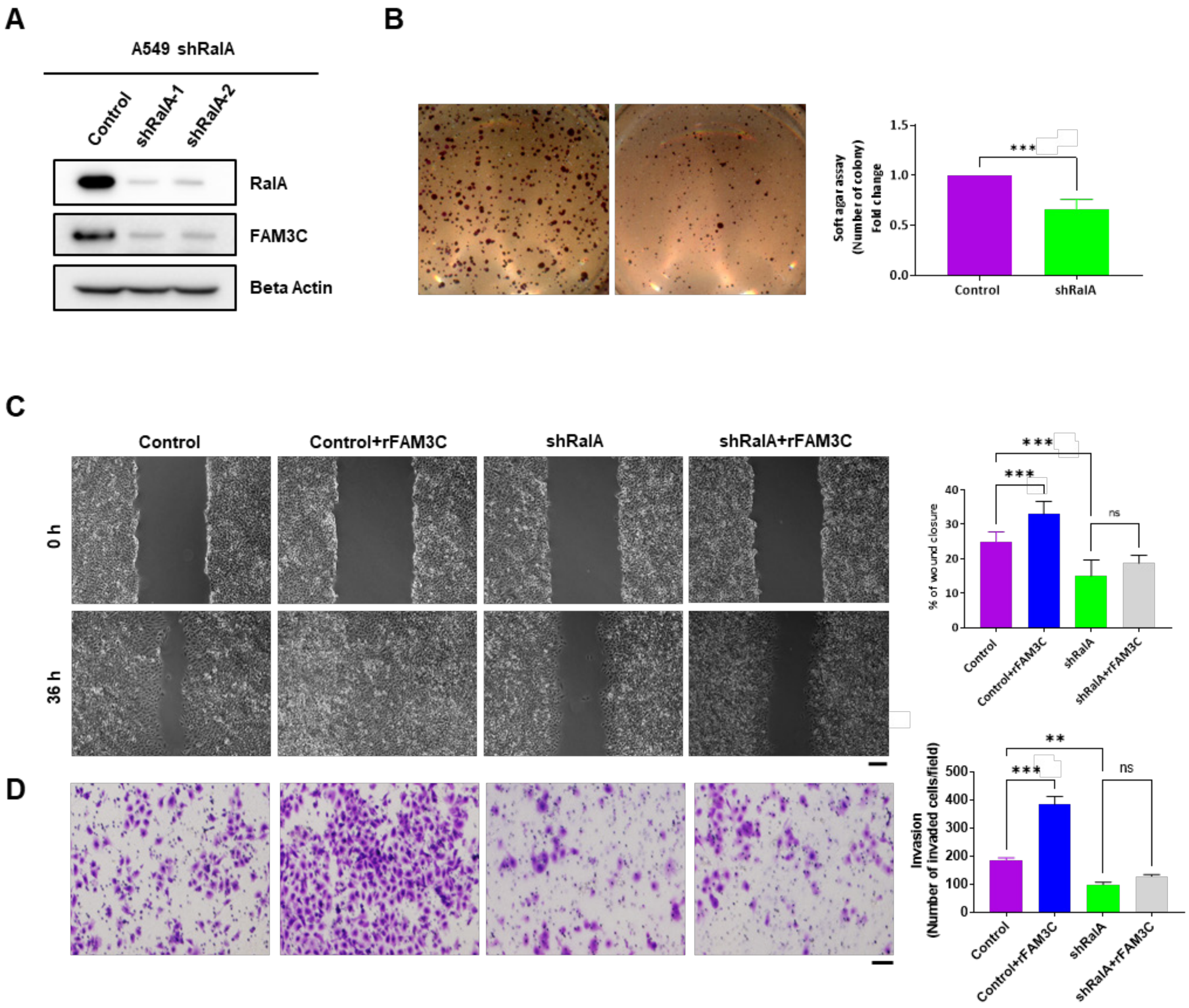

Figure S8. RalA knockdown decreases cell motility and colony formation in A549 lung carcinoma cells. RalA knockdown A549 cell line was generated through shRNA transfection. **(A)** Knockdown of RalA decreases expression of FAM3C. **(B)** Soft agar colony forming assay upon RalA knockdown in A549 lung carcinoma cells. **(C-D)** Representative images are shown. Scale bar: 200 μM. The cell motility of FAM3C knockdown cells was examined by wound healing **(C)** and matrigel-coated transwell assay **(D)**. A549 control and RalA knockdown (shRalA) cells were treated with human recombinant FAM3C (rFAM3C) protein and the effect of rFAM3C on cell motility was examined by wound healing and Matrigel invasion assays. The data presented in right panels of (c-d) are the results of three independent experiments. Data presented as mean ± SEM, ns: not significant, ** $p < 0.01$ and *** $p < 0.001$.

Figure S9

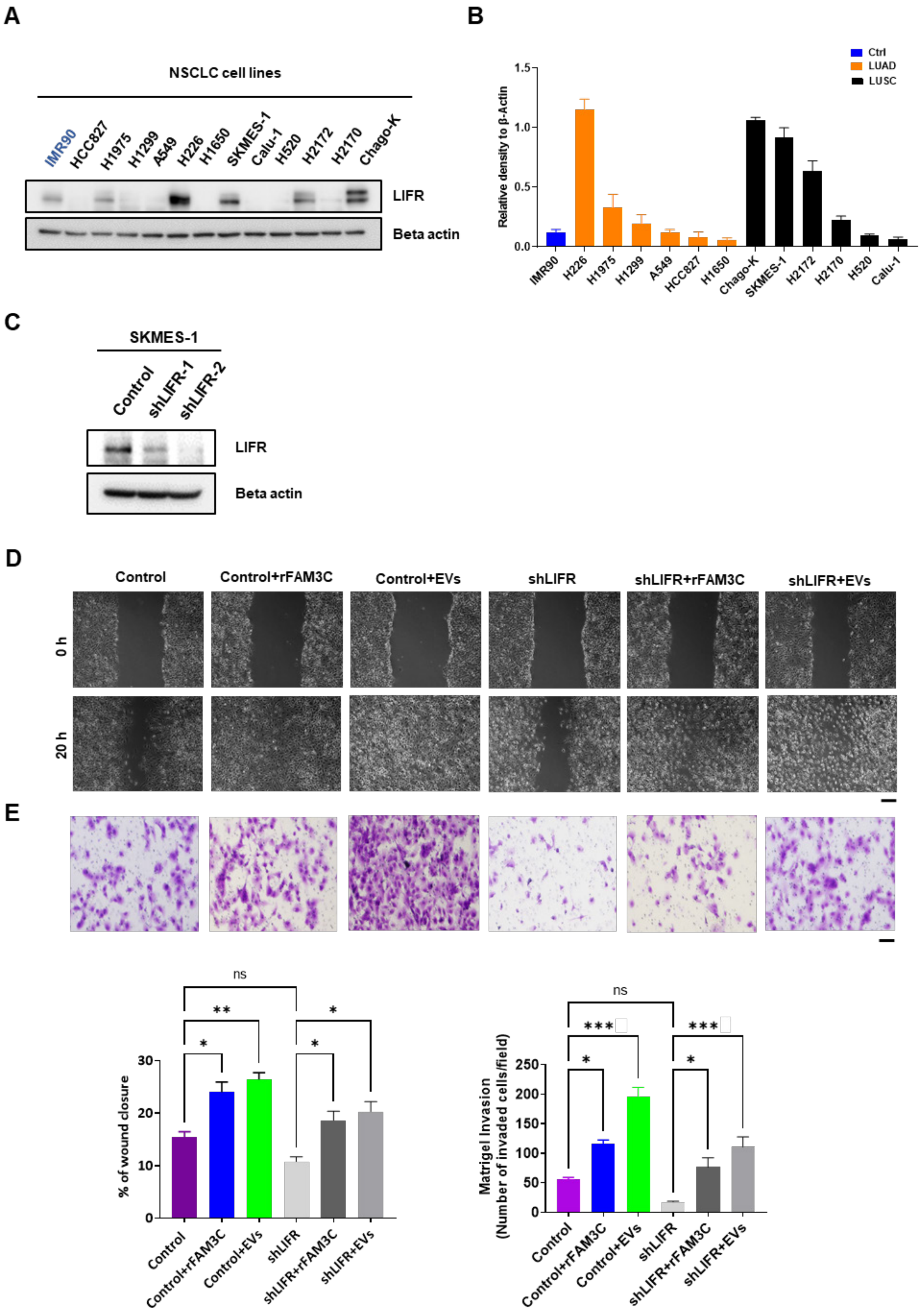

Figure S9. EVs from SKMES-1 cells and rFAM3C induce the invasive activity in control and LIFR_knockdown cells. **(A)** Western blot analysis of LIFR in 12 NSCLC cell lines compared to normal lung

fibroblast (NLF) cell line (IMR90). **(B)** The histogram represents expression levels of LIFR in normal lung fibroblast (blue), LUAD (orange) and LUSC (black) after normalization against β-actin. **(C)** LIFR knockdown SKMES-1 cell line was generated through shRNA transfection and confirmed by western blot analysis. **(D-E)** Representative images are shown. Scale bar: 200 μM. The cell motility of LIFR control and knockdown cells with and without recombinant FAM3C (rFAM3C) and extracellular vesicles (EVs) treatment was examined by wound healing **(D)** and Matrigel-coated transwell assay **(E)**. The data presented in histograms of **(B, D, E)** are the results of three independent experiments. Data presented as mean ± SEM, ns: not significant, * $p < 0.5$ and ** $p < 0.01$.

Figure S10

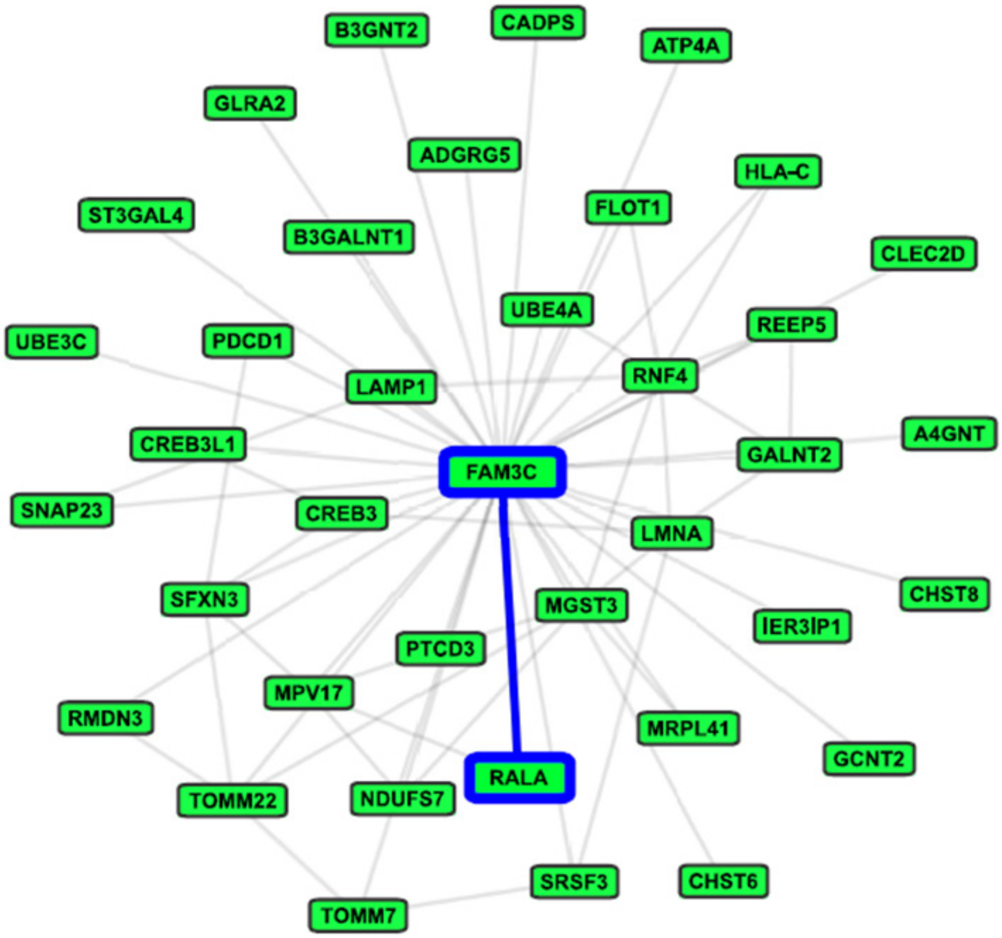

Figure S10. Pattern diagram of biological network integration of genes positively correlated to FAM3C was generated using GPS-Prot database.

3) Supplementary Tables

**Table S1. Top 20 of Log2 fold change EV proteins in Tumour cells versus normal fibroblast cells**				
**Accession**	**GS**	**Gene Name**	**Log2 fold change (Tumour/IMR90)**	***p* value**
Q1KLZ0	PS1TP5BP1	Q1KLZ0_HUMAN PS1TP5-binding protein 1 OS=Homo sapiens GN=PS1TP5BP1 PE=2 SV=1	11.36085762	0.049628
Q8IZ29	TUBB2C	Q8IZ29_HUMAN Tubulin, beta 2C OS=Homo sapiens GN=TUBB2C PE=2 SV=1	8.667404994	0.009599
Q9BVA1	TUBB2B	TBB2B_HUMAN Tubulin beta-2B chain OS=Homo sapiens GN=TUBB2B PE=1 SV=1	8.268796133	0.000671
P62258	YWHAE	1433E_HUMAN 14-3-3 protein epsilon OS=Homo sapiens GN=YWHAE PE=1 SV=1	7.935795981	0
P05023	ATP1A1	AT1A1_HUMAN Sodium/potassium-transporting ATPase subunit alpha-1 OS=Homo sapiens GN=ATP1A1 PE=1 SV=1	7.295494419	0
Q6LES2	ANXA4	Q6LES2_HUMAN Proliferation-inducing protein 28 (Fragment) OS=Homo sapiens GN=ANXA4 PE=2 SV=1	7.091237886	0.000151
P16422	EPCAM	EPCAM_HUMAN Epithelial cell adhesion molecule OS=Homo sapiens GN=EPCAM PE=1 SV=2	7.078244026	0.001095
Q5JQ44	RAP2B	Q5JQ44_HUMAN Putative uncharacterized protein DKFZp547A0616 (Fragment) OS=Homo sapiens GN=DKFZp547A0616 PE=2 SV=1	7.013429907	0.001449
B4E3B6	HSPA1A	B4E3B6_HUMAN cDNA FLJ54408, highly similar to Heat shock 70 kDa protein 1 OS=Homo sapiens PE=2 SV=1	7.005043055	2.4E-05
B3KQ25	PSME3	B3KQ25_HUMAN cDNA FLJ32659 fis, clone TESTI1000045, highly similar to Proteasome activator complex subunit 3 OS=Homo sapiens PE=2 SV=1	6.981036669	0
A9C4C1	RPS9	A9C4C1_HUMAN Ribosomal protein S9 OS=Homo sapiens GN=RPS9 PE=3 SV=1	6.948057484	1.24E-07
B7Z9U6	RDX	B7Z9U6_HUMAN cDNA, FLJ78960, highly similar to Radixin OS=Homo sapiens PE=2 SV=1	6.894240108	5.22E-15
P61006	RAB8A	RAB8A_HUMAN Ras-related protein Rab-8A OS=Homo sapiens GN=RAB8A PE=1 SV=1	6.76456763	0
B4DRR0	KRT6A	B4DRR0_HUMAN cDNA FLJ53910, highly similar to Keratin, type II cytoskeletal 6A OS=Homo sapiens PE=2 SV=1	6.615428208	0
B3KTQ2	MFGE8	B3KTQ2_HUMAN cDNA FLJ38589 fis, clone HCHON2010074, highly similar to LACTADHERIN OS=Homo sapiens PE=2 SV=1	6.475836425	0.026288
Q92520	FAM3C	FAM3C_HUMAN Protein FAM3C OS=Homo sapiens GN=FAM3C PE=1 SV=1	6.433849927	5.83E-09
P61020	RAB5B	RAB5B_HUMAN Ras-related protein Rab-5B OS=Homo sapiens GN=RAB5B PE=1 SV=1	6.421924326	0
Q9P2B2	PTGFRN	FPRP_HUMAN Prostaglandin F2 receptor negative regulator OS=Homo sapiens GN=PTGFRN PE=1 SV=2	6.381945879	0.000249
Q6FG99	RPLP1	Q6FG99_HUMAN RPLP1 protein OS=Homo sapiens GN=RPLP1 PE=4 SV=1	6.362466894	1.15E-09

Table S2. The expression of FAM3C in NSCLC tissue array (n=100)

Sample		N	FAM3C		
Type	Pathological diagnosis		Positive	Negative	Positive rate (%)
Malignant	NSCLC lung cancer tissue	45	44	1	98
Normal	Cancer adjacent normal lung tissue	47	17	30	36
Normal	Normal lung tissue	8	0	8	0

Table S3. qPCR primers used in this study

Gene	*Forward primer (5' - 3')*	*Reverse primer (5' - 3')*
**ITGB3**	CGCTAAATTTGAGGAAGAACG	GAAGGTAGACGTGGCCTCTTT
**SNAI2**	CGAACTGGACACACATACAGTG	CTGAGGATCTCTGGTTGTGGT
**TGM2**	ACTACAACTCGGCCCATGAC	TGGTCATCCACGACTCCAC
**VIM**	AAGAGATGGCTCGTCACCTT	GGGTGTCAACCAGAGGAAGT
**GLIPR1**	AGTTCCGATCAGAGGTGAAACC	GCTTCAGCCGTGTATTATGTGA
**TIMP3**	AGCTTCCGAGAGTCTCTGTG	CACCTCTCCACGAAGTTGC
**TMEM158**	GCCCTTTCCCCTCTATTGC	TCATTTTCTGCCATGAATCTAGG
**MFAP5**	GCATCGGCCGGTTAAACAAT	TCACAGGGAGGAAGTCGGAA
**SPOCK1**	CAACTGCTTGTTCCCAGAGG	GCCAATGACTTCCCTATCCA
**GAPDH**	ACATCGCTCAGACACCATG	TGTAGTTGAGGTCAATGAAGGG

Table S4. shRNA sequences used in this study

shRNA	*Sequences*
**hRALA (shRNA#1)**	AGTTATTGGCATGGTTGTTGCATATAGTTAAACTGAGAGTA
**hRALA (shRNA#2)**	TGCTAGATGGGGAGGAAGTCCAGATCGATATCTTAGATACA
**hLIFR (shRNA#1)**	TGTTGAGGATACTTCTGCAGATTCGATATTAGTAAAATGGG
**hLIFR (shRNA#2)**	AGTGATACTTGTAGGCTCAGACATAACATTTTGTTGTGTGA
